# Canagliflozin alleviates progestin resistance by suppressing RARβ/CRABP2 signaling in THRB knockout endometrial cancer cells

**DOI:** 10.3389/fphar.2025.1573032

**Published:** 2025-04-30

**Authors:** Ye Yang, Jieyun Zhou, Qiaoying Lv, Qicheng Ni, Baichun Hu, Yulong Wang, Shuning Qu, Guoting Li, Wenjie Yang, Ruihua Zhong, Xiaojun Chen, Yan Zhu

**Affiliations:** ^1^ School of Pharmacy, Fudan University, Shanghai, China; ^2^ Laboratory of Reproductive Pharmacology, NHC Key Lab of Reproduction Regulation, Shanghai Engineering Research Center of Reproductive Health Drug and Devices, Shanghai Institute for Biomedical and Pharmaceutical Technologies, Shanghai, China; ^3^ Department of Gynecology, Obstetrics and Gynecology Hospital of Fudan University, Shanghai, China; ^4^ Key Laboratory of Structure Based Drug Design and Discovery, Shenyang Pharmaceutical University, Shenyang, China; ^5^ Shanghai Tenth People’s Hospital, School of Medicine, Tongji University, Shanghai, China

**Keywords:** endometrial cancer, thyroid hormone receptor β, canagliflozin, medroxyprogesterone acetate, progestin resistance, retinoic acid receptor β

## Abstract

**Introduction:**

Progestin resistance has emerged as a significant barrier to the conservative management of endometrial cancer (EC). The mechanisms underlying progestin resistance in endocrine therapy remain incompletely understood. Previous studies have suggested that silencing thyroid hormone receptor B (THRB) is associated with progestin resistance in EC cells.

**Methods:**

THRB-knockout RL95-2 (THRB^(−/−)^/RL95-2) cells were constructed to investigate progestin resistance mechanisms. Cell proliferation and apoptosis were assessed in RL95-2 and THRB^(−/−)^/RL95-2 cells treated with canagliflozin (CANA), medroxyprogesterone acetate (MPA), and their combination using CCK-8, EdU, and flow cytometry assays. *In vivo*, nude mouse xenograft models were used to evaluate the efficacy of CANA and MPA. Transcriptomic and proteomic analyses were performed to identify pathways associated with progestin resistance. Molecular dynamics simulations, along with western blotting and immunohistochemistry were utilized to validate the targets of CANA. Electrophoretic mobility shift assays and dual luciferase reporter assays were employed to investigate the interactions between TRβ, RARβ, and CRABP2.

**Results:**

THRB^(−/−)^/RL95-2 cells were successfully constructed. CANA demonstrated a strong binding affinity for TRβ. Both MPA and CANA suppressed proliferation in RL95-2 cells, but MPA was ineffective in THRB^(−/−)^/RL95-2 cells, indicating that THRB deficiency induced progestin resistance. CANA significantly inhibited proliferation and promoted apoptosis in THRB^(−/−)^/RL95-2 cells. *In vivo*, CANA, either alone or in combination with MPA, significantly reduced tumor growth in xenograft models derived from both wild-type and THRB-knockout RL95-2 cells. Transcriptomic and proteomic analyses revealed that progestin resistance in EC was linked to the retinoic acid signaling pathways. Western blotting confirmed that the expressions of RARβ, RXRA and CRABP2 were significantly elevated in THRB^(−/−)^/RL95-2 cells. Treatment with CANA, alone or in combination with MPA, effectively reduced the expression of these proteins. Immunohistochemical analysis demonstrated that RARβ expression was significantly increased in uterine tissues from patients with progestin-insensitive EC or endometrial atypical hyperplasia. Electrophoretic mobility shift assays and dual luciferase reporter assays demonstrated that TRβ negatively modulated RARβ expression by binding to its promoter, while RARβ positively regulated CRABP2 expression.

**Conclusion:**

THRB knockout activated retinoic acid pathway, leading to progestin resistance. CANA targeted RARβ and RXRA, downregulated CRABP2, restored BAX levels, and counteracted progestin resistance. The combination of CANA and MPA presented a novel strategy for alleviating progestin resistance and enhancing clinical efficacy.

## Graphical Abstract

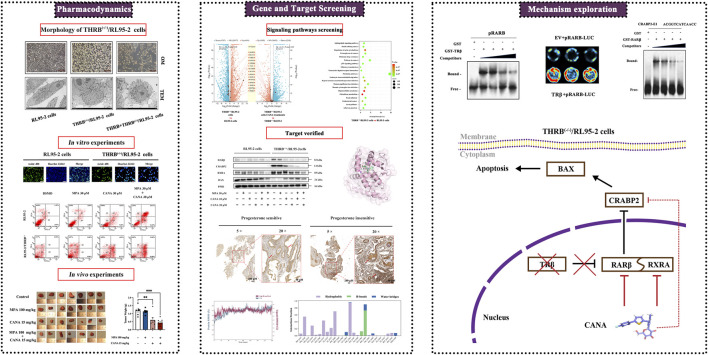

## Highlights

• THRB deficiency was demonstrated to correlate with progestin resistance in RL95-2 endometrial cells.
• Knockout THRB was found to activate retinoic acid signaling pathway, driving the development of progestin resistance in RL95-2 cells.
• Canagliflozin effectively counteracted progestin resistance in THRB-knockout RL95-2 cells by targeting RARβ, downregulating the expression of CRABP2.
• Canagliflozin in combination with medroxyprogesterone acetate is likely a potentially effective regimen for the treatment of endometrial cancer and counteracts progestin resistance.

## 1 Introduction

Globally, nearly 420,000 new cases of endometrial cancer (EC) are reported annually, with increasing incidence and mortality rates (Siegel et al., 2024). In China, the National Cancer Center estimated 84,520 new cases and 17,523 deaths from EC in 2022 (Han et al., 2024). Despite the latest molecular classification of EC (Crosbie et al., 2022), ECs have traditionally been classified into two types based on histology and clinical outcomes. Conventionally, Type I EC, constituting the vast majority of ECs, is predominantly endometrioid adenocarcinomas (Setiawan et al., 2013), and is associated with chronic estrogenic stimulation and lack of progestin antagonism. In contrast, type II EC is negative for estrogen and progestin receptors (Baker-Rand and Kitson, 2024).

Treatment options for EC depend on the patient’s condition and disease stage (Baker-Rand and Kitson, 2024; Ronsini et al., 2024). Currently, more women with reproductive age would like to opt for non-surgical treatments to preserve fertility (Suzuki et al., 2024). Consequently, hormone therapy remains a cornerstone for the hormone receptor-positive EC, especially in early and recurrent cases (Suzuki et al., 2024). Therapy with progestins alone had a success rate of 50%–80% in the treatment of early EC. This means that a significant proportion of patients fail to respond to therapy due to progestin resistance (Park et al., 2012; Ma et al., 2021a; Tronconi et al., 2022; Suzuki et al., 2024). Despite recent studies suggesting that combining MPA with metformin possibly antagonized progestin resistance in EC (Mitsuhashi et al., 2016), no consensus has been achieved, and there is still a need to find more effective therapeutic options (Chae-Kim et al., 2021).

Mechanisms of hormone resistance in EC included downregulation or mutation of progestin receptors, activation of the PI3K/Akt/mTOR, MAPK, and Wnt/β-catenin signaling pathways, and reliance on alternative signaling pathways (Li et al., 2019; Ding et al., 2021; Ma et al., 2021b; Abdelmaksoud et al., 2023; Liu et al., 2023). Accordingly, several targeted inhibitors have been developed, such as Everolimus and Alpelisib. However, these compounds could induce hyperglycemia, particularly in diabetic or prediabetic patients, limiting their clinical use (Passarelli et al., 2022; Slomovitz et al., 2022). Therefore, there is an urgent need to develop better therapeutic strategies (Ma et al., 2021a). The exploration of novel therapies depends on the identification of new signaling and pathways driving EC resistance. Current progestin-resistant cell models have been established via treating Ishikawa cells with prolonged and low-dose progestin (e.g., Medroxyprogesterone Acetate, MPA) (Zhang et al., 2011; Ma et al., 2021b). However, these cells would be influenced by the culture environment and the cellular status, which could change over time, possibly resulting in unreliable results.

Thyroid hormone receptors (TRs), widely expressed in multiple tissues and regulate development and differentiation, belonging to nuclear receptors and are classified into two subtypes—thyroid hormone receptor α (TRα) and thyroid hormone receptor β (TRβ) (Cusi, 2024; Spinelli et al., 2024). Emerging evidence suggested that TRβ could act as a tumor suppressor in some solid tumors such as hepatocellular carcinoma, breast cancer, ovarian cancer, renal cell carcinoma, and thyroid cancer (Bolf et al., 2021; Davidson et al., 2021; Zhi et al., 2024; Zhu and Cheng, 2024). Additionally, TRβ was considered as a regulatory agent of lipid metabolism as well. Recently, a novel agonist targeting TRβ has been approved to regulate cholesterol metabolism and treat hyperlipidemia. Previously, we found that TRβ exhibited a tumor-suppressive role in EC cells and silencing THRB obviously diminished the inhibitory effects of MPA on the proliferation of RL95-2 EC cells, whereas silencing THRA did not affect the growth of the cells. TRβ likely took a critical role in driving drug resistance in EC hormonal therapy by activating the mTOR signaling pathway, which was considered one of mechanisms inducing progestin resistance (Ren et al., 2022).

In the study, THRB knockout RL95-2 (THRB^(−/−)^/RL95-2) cells were constructed for further investigating the role of TRβ in the growth of the EC cells and modulation of MPA sensitivity. Canagliflozin (CANA) was assessed in wild-type and THRB^(−/−)^/RL95-2 cells because it demonstrated a strong ability to interact with TRβ through molecular docking and virtual screening. Nude mice with xenograft tumors were used to evaluate anti-tumor effects. Additionally, both transcriptomics and proteomics were utilized to probe the potential targets of CANA. Molecular dynamics simulations were conducted to predict the interactions between CANA and candidate targets. Finally, electrophoretic mobility shift and dual luciferase reporter assay were employed to reveal the interaction modes among the candidate targets in THRB^(−/−)^/RL95-2 cells.

## 2 Materials and methods

### 2.1 Reagents

Medroxyprogesterone Acetate (MPA) was gifted by Xianju Pharmaceutical Co., Ltd (Taizhou, Zhejiang, China). Canagliflozin (CANA), acarbose, atorvastatin and LE135 were purchased form MCE (Monmouth Junction, United States).

### 2.2 Structure superposition and protein contacts atlas analysis

The crystallographic structures of TRα (PDB code: 3JZB (Martínez et al., 2009)) and TRβ (PDB code: 7WMO (Li et al., 2022a)) which downloaded by using the GetPDB function in Maestro 9.0 from RCSB PDB Data Bank. The structures and sequences were performed to assess the similarity of amino acid residues and the congruence of their spatial folding using the Discovery Studio 3.0 software package, respectively (Karunagaran et al., 2017). During the structural superposition process, each co-crystal ligand was anchored to its respective TR active sites. The superimposed structures and data were subsequently exported and compiled for comprehensive analysis. Protein contact atlas analysis was conducted to investigate the crystal TRα/β protein-ligand interactions and identified key protein residues essential for molecular binding, utilizing the online resource available at website of https://www2.mrc-lmb.cam.ac.uk/(Kayikci et al., 2018).

### 2.3 Virtual screening and molecular docking

The structure of TRβ (PDB code: 7WMO) and RARβ (PDB code: 1XAP) were utilized for virtual screening and molecular docking, respectively. Candidate ligands to match TRβ were downloaded from the public Zinc database authorized by the Food and Drug Administration (FDA) at the website https://zinc15.docking.org/catalogs/fda/(Irwin and Shoichet, 2005) and put into Schrödinger software (Fratev et al., 2018). The preparation of protein and ligand structures were performed as previously described (Yang et al., 2021a). Docking investigations were carried out employing the Glide module within the Schrödinger package (Friesner et al., 2004). Initially, receptor grids were generated at the binding sites of TRβ, delineated by the location of the co-ligand within a 20 Å radius. The setting of virtual screening mode of action restriction was based on the intrinsic binding mode of TRβ agonists (Li et al., 2022b) to increase the selective strength of action of the compounds. Multiple docking score functions, including the docking score, glide gscore, XP gscore and MM GBSA ∆G _Bind_ were calculated to evaluate the binding affinity between the ligands and receptors (Halgren et al., 2004).

### 2.4 Cell culture and treatments

Human RL95-2 and AN3CA EC cell lines were purchased from the American Type Culture Collection (ATCC, Manassas, VA, United States), and the KLE cell lines were purchased from the China Center for Type Culture Collection. 293 T cell lines were purchased by Nanjing Ruigan Biotechnology Co., Ltd. RL95-2, KLE and 293 T cells were cultured in Dulbecco’s Modified Eagle Medium/F12 (DMEM/F12; Gibco, Carlsbad, CA, United States), and AN3CA cells were cultured in McCoy’s 5A medium and Minimum Essential Medium (Gibco, Waltham, MA, United States), respectively. All culture media were supplemented with 10% fetal bovine serum (FBS; Gibco, Auckland, New Zealand).

THRB^(−/−)^/RL95-2 cells were generated by knocking out of the THRB gene in RL95-2 cells using CRISPR technology (Cyagen Biosciences, Suzhou, China). After reintroducing the THRB plasmid was into the THRB^(−/−)^/RL95-2 cells to restore the expression of thyroid hormone receptor beta (TRβ), these cells were referred to as THRB + THRB^(−/−)^/RL95-2. All cells were incubated at 37°C in a humidified atmosphere with 5% CO_2_ and sub-cultured after reaching 80% confluence.

In the assays of cell viability, RL95-2, THRB^(−/−)^/RL95-2, KLE, and AN3CA cells were seeded in 96-well plates at a density of 8 × 10^3^ cells per well. The cells were treated with DMSO, MPA, CANA, acarbose, atorvastatin, or triiodothyronine (T3) at varying concentrations for 48 h. Then, 10 μL of cell counting kit-8 (CCK-8) solution (CK04, Dojindo Laboratories, Kumamoto, Japan) was added to each well, and optical density (OD) was measured at 450 nm using a microplate reader (BioTek ELX-800). Cell viability (%) was calculated using the formula: cell viability (%) = (OD _Test_/OD _Control_) × 100%. The viable cell inhibition rate and IC_50_ calculation were detailed in this work of ours (Cao et al., 2019; Zhu et al., 2019).

In other experiments *in vitro,* RL95-2 and THRB^(−/−)^/RL95-2 cells were cultured for 24 h after being sub-cultured and then treated with DMSO, MPA (30 μM), CANA (10 μM or 30 μM), and combinations of MPA and CANA (10 μM or 30 μM) for an additional 48 h.

### 2.5 Animals

Fifty-six female athymic nude mice of 18 ± 1 g were purchased from Beijing Vital River Laboratory Animal Technology Co., Ltd (Beijing, China) and housed under specific-pathogen-free conditions in individually ventilated cage animal facility at 25°C with 70% humidity and a 12 h dark/light cycle. Mice were treated in compliance with guidelines of the Institutional Animal Care and Use Committee of Shanghai Institute for Biomedical and Pharmaceutical Technologies (Animal Experiment Ethics Approval No. 2022–31) The mice were housed 4 or 5 per cage with free access to sterilized tap water and standard chow. Prior to the experiment, the mice were fed for 1 week’s adaption.

### 2.6 Collection of endometrial tissues

This study was approved by the Ethics Committee of the Shanghai Institute for Biomedical and Pharmaceutical Technologies and the approval number was PJ 2022-33. Endometrial tissues were obtained from patients admitted to the Obstetrics and Gynecology Hospital of Fudan University, including 8 patients with endometrial atypical hyperplasia (EAH) and 12 patients with endometrial carcinoma (ages 21–39), who underwent hysteroscopy between December 2018 and December 2019. Among them, the progestin-insensitive group included 3 samples of EAH and 4 of EC; while 5 samples of EAH group and 8 of EC were progestin-sensitive. Biopsied uterine tissues from patients with adenocarcinoma (AC) and EAH, either sensitive or insensitive to progestin therapy (including MPA, Mirena^®^, or megestrol acetate), were fixed in formalin and embedded in paraffin. Progestin resistance was defined as disease progression at any time during treatment, stable disease after 7 months of treatment, or lack of complete response (CR) after 10 months of treatment. Patients achieving CR within 10 months were classified as progestin sensitive.

### 2.7 Detection of cell proliferation and apoptosis

The RL95-2 and THRB^(−/−)^/RL95-2 cells were analyzed quantitatively using the EdU proliferation assay. Cells were seeded into six-well culture plates at a uniform density of 5 × 10^5^ cells per well. After a 24-h incubation, the cells were treated with DMSO, MPA, CANA or MPA combined CANA. Following an additional 48-h culture, the cells were stained with 10 µM EdU using the kFluor488-EdU Cell Proliferation Detection Kit (C0071S, Beyotime, Shanghai, China), according to the manufacturer’s instructions. After a 2-h incubation with EdU, proliferative activity was visualized using a Leica fluorescence microscope (Leica Microsystems, Wetzlar, Germany). The percentages of EdU-labeled cells and Hoechst 33,342-labeled cells were quantified using ImageJ software (Rawak Software, Stuttgart, Germany).

For apoptosis detection, RL95-2 and THRB^(−/−)^/RL95-2 cells were cultured in six-well plates at a density of 1 × 10^6^ cells per well and treated with MPA, CANA and their combination. Following 48 h incubation, the cells were stained with FITC-conjugated Annexin V and propidium iodide PI using the Annexin V-FITC Apoptosis Detection Kit (C1062M, Beyotime, Shanghai, China), according to the manufacturer’s protocol. The apoptotic cell percentages were quantified *via* flow cytometry using a FACSVantage SE system (BD Biosciences, San Jose, CA, United States).

### 2.8 Transmission electron microscopy (TEM)

Cell morphological changes were observed by using electron microscopy. Briefly, RL95-2, THRB^(−/−)^/RL95-2 and THRB + THRB^(−/−)^/RL95-2 cells were fixed in electron microscopy fixative (G1102, Servicebio, Wuhan, China) containing 1% osmium tetroxide (8,456, Ted Pella Inc., Redding, CA, United States) the cells were embedded osmotically in acetone with an 812 embedding agent (90,529–77–4, SPI, West Chester, PA, United States), and the experimental approaches referred to Zhu et al. (2010).

### 2.9 Xenograft tumor in nude mice

A xenograft tumor assay in BALB/c nude mice was performed to evaluate the therapeutic efficacy of MPA and CANA and their combination. After acclimating to the environment for 1 week, the nude mice were injected subcutaneously with 200 μL of a suspension containing 5 × 10^6^ cells/mL of RL95-2 or THRB^(−/−)^/RL95-2 cells on the right flank. When tumors reached approximately 100 mm^3^ in volume, the mice were randomly assigned to four groups (7 mice per group) and treated via gastric perfusion with either a solvent control, MPA (100 mg/kg), CANA (15 mg/kg), or a combination of MPA (100 mg/kg) and CANA (15 mg/kg) for 4 weeks. The experimental doses of MPA and CANA were converted from the clinical dose used in humans to nude mice based on body surface area coefficient (Chen et al., 2015; Tamauchi et al., 2018; Yamagami et al., 2018). Tumor volumes and body weights were measured twice a week using the formula: V (mm^3^) = 0.5×(L × W × W), where V represents tumor volume, L is length, and W is width (Cao et al., 2019). At the end of 28 days’ treatment, all mice were anesthetized with 3% pentobarbital sodium, and tumors were surgically excised for weighing, measurement, imaging and frozen.

### 2.10 Transcriptomics and proteomics analysis

In assays of transcriptomics, RL95-2 cells were treated with DMSO and 30 μM CANA for 48 h, while THRB^(−/−)^/RL95-2 cells were treated with DMSO, 30 μM MPA, and 30 μM CANA. The methods of RNA extraction and quality control, high-throughput sequencing, and data statistics were consistent with our previous studies (Ren et al., 2022). Differentially expressed genes (DEGs) were identified based on the criteria |log2FC| ≥ 1 and *P*-value ≤0.05. DEGs were compared among the following groups: DMSO treated THRB^(−/−)^/RL95-2 cells versus RL95-2 cells, THRB^(−/−)^/RL95-2 cells with 30 μM CANA treatment versus DMSO, and THRB^(−/−)^/RL95-2 cells with 30 μM MPA treatment versus DMSO. Identified DEGs were subjected to the Kyoto Encyclopedia of Genes and Genomes (KEGG) signaling pathway and disease enrichment analysis. KEGG functional analysis included annotation and classification of pathways associated with these genes. The final data were from three independent experiments.

In assays of proteomics, RL95-2 and THRB^(−/−)^/RL95-2 cells were treated with either DMSO or 30 μM CANA for 48 h. Then, the samples were prepared *via* protein extraction, denaturation, reduction, alkylation, tryptic digestion, and peptide cleanup. The commercially available iST Sample Preparation Kit (PreOmics, Germany) was used according to the manufacturer’s protocol. The nanoElute 2 liquid chromatography system (Bruker Daltonik, Bremen, Germany) was coupled to the timsTOF Pro2, an ion-mobility spectrometry quadrupole time-of-flight mass spectrometer (Bruker Daltonik, Bremen, Germany). Data-Independent Acquisition (DIA) data acquisition was performed in diaPASEF mode. Twenty-two precursor isolation windows (40 Th each) were defined, spanning m/z 349 to 1,229. Raw DIA data were processed and analyzed using Spectronaut 18 (Biognosys AG, Switzerland) with default settings. *Homo sapiens* database (version 2022, 20,610 entries) was downloaded from UniProt. Trypsin was specified as the digestion enzyme with specificity for the digest type. Carbamidomethylation on cysteine was set as a fixed modification, while oxidation on methionine and acetylation at the protein N-terminus were set as variable modifications. Retention time prediction was set to dynamic iRT. Data extraction was automatically determined by Spectronaut, leveraging extensive mass calibration. The Q-value (FDR) cutoff was set at 1% for both precursor and protein levels. Decoy sequences were generated using the “mutated” option, which introduces a random number of amino acid position swaps (minimum = 2, maximum = length/2). The normalization strategy was set to local normalization. Peptides passing the 1% Q-value cutoff were used for major group quantification using the MaxLFQ method. Different protein enrichment and group comparisons were performed similarly to transcriptomic profiling.

Enrichment results were visualized using an online tool (http://www.bioinformatics.com, accessed on 24 June 2024 and 11 December 2024).

### 2.11 Molecular dynamics simulation

Molecular dynamics simulations were conducted using Desmond (v3.8) within Schrödinger software to analyze the optimal conformation of the RARβ/CANA complex obtained from docking studies. The methodology for molecular dynamics simulations, trajectory analysis, and molecular mechanics/generalized Born surface area (MM-GBSA) calculations followed the protocol outlined in our previous work (Yang et al., 2021a). In this study, the simulation duration was extended from 100 ns to 200 ns to provide a more detailed characterization of the complex’s mode of action in a simulated aqueous environment.

### 2.12 Immunohistochemistry analyze

Immunohistochemistry was utilized to evaluate the expression and the site of RARβ in human uterine tissues. The sections were baked in a thermostat at 58°C for 1.5 h and then deparaffinized using standard procedures. Performances were conducted in accordance with the manufacturer’s instructions. The sections were incubated overnight with the primary antibody at 4°C in a humidified chamber and then stained using DAB working solution (AR1027-9, BioDee, Beijing, China). Finally, the sections were dehydrated, sealed, and imaged under a microscope. RARβ expression in each section was quantified using ImageJ 1.48 (Rawak Software, Stuttgart, Germany) with the IHC Tools plugin, based on five random fields of view at ×20 magnification. The anti-RARβ antibody (1:200, 14013-1-AP) was purchased from Proteintech, Inc (Wuhan, China).

### 2.13 Plasmid transfection

The THRB^(−/−)^/RL95-2 cells were seeded into six-well plates at a density of 2 × 10^6^ cells/well and incubated for 24 h. When the cells reached the confluence of 70%–90%, transfection was carried out. The 2,500 ng of THRB plasmid (GenePharma, Shanghai, China) and P3000 were mixed with Lipo3000 (Invitrogen, Waltham, MA, United States) diluted in Opti-MEM (Thermo Scientific, Waltham, MA, United States). The plasmid and Lipo3000 mixture were then added to the six-well plates and incubated for 48 h for subsequent Western blot analysis. The plasmid-backfilled cell line was named THRB + THRB^(−/−)^/RL95-2 cells.

### 2.14 Electrophoretic mobility shift assay (EMSA)

The recombinant plasmids for TRβ and RARβ were constructed using the pGEX-6P-1 vector, and the experiments were carried out in Nanjing RuiGan Biotechnology Co., Ltd. (Nanjing, China). Single colonies of *E. coli* transformed with the recombinant plasmids were selected and inoculated into 2 mL of LB liquid medium. The cultures were incubated overnight at 37°C and 220 rpm. Endotoxin-free plasmids were extracted using the PureLink™ Expi Endotoxin-Free Plasmid Purification Kit (A33073, Thermo Fisher Scientific). Monoclonal colonies were further cultured in 5 mL of LB medium until the OD_600_ reached 0.5 at 37°C. Protein expression of TRβ and RARβ were induced at 16°C overnight. After induction, the cultures were centrifuged, and the bacterial pellets were lysed. The supernatant was collected, and its protein concentration was measured and analyzed. The protein-containing supernatant was filtered twice through a 0.4 μm membrane and purified using the AKTA avant protein purification system (Cytiva) to enrich GST-TRβ and GST-RARβ protein while removing excess salts.

Biotin-16-UTP was conjugated with RARβ and CRABP2 promoter DNA *in vitro*, following the protocol of the DIG DNA Labeling and Detection Kit (3,353,575,910, Roche, F. Hoffmann-La Roche AG, Basel, Switzerland). Briefly, 2 μg of TRβ or RARβ protein was incubated with 4 μg of biotin-labeled RARβ and CRABP2 promoter DNA in EMSA binding buffer at 4°C for 30 min. EMSAs were performed with the DIG Gel Shift Kit according to the manufacturer’s instructions, and signals were detected using the provided chemiluminescent substrate. For competition assays, 10-fold, 50-fold, and 100-fold excess unlabeled competitor DNA probes were added to the reactions (lines 3, 4, and 5, respectively). The resulting complexes were separated by acrylamide gel electrophoresis under conditions similar to Western blotting, using GST-specific commercial antibodies (1:5,000, M20007, Abmart, Shanghai, China).

### 2.15 Dual luciferase reporter assay

For the dual-luciferase assay, 293 T cells were used and seeded in 24-well plates and then co-transfected with 100 ng of THRB promoter reporter plasmid using Lipofectamine 3,000 (Thermo Fisher Scientific, United States) in Opti-MEM (Gibco, Thermo Fisher Scientific, United States) after the confluence of the cells reached to 80%. The plasmids were performed by Nanjing RuiGan Biotechnology Co., Ltd. (Nanjing, China). After being cultured for 48h, the cells were harvested. Dual Luciferase Reporter Assay Kit (DL101-01, Vazyme, Nanjing, China) kit was utilized to perform the dual fluorescence reporter assay. Performances were conducted following the manufacturer’s instruction. The fluorescence signal value of LUC using a multilabel plate reader (BioTek, Beijing, China). Then add 100 μL of the termination solution and RLUC luminescent substrate in the kit, and measure the fluorescence signal value of RLUC using a multilabel plate reader (BioTek, Beijing, China). The relative fluorescence intensity was the value of Firefly Luciferase (LUC)/Renilla Luciferase (RLUC).

### 2.16 Western blotting

RL95-2 and THRB^(−/−)^/RL95-2 cells were cultured for 24 h, followed by treatment with DMSO, MPA, and CANA and their combination for 48 h. The performances of Western blotting were conducted according to previous references (Ren et al., 2022). The antibodies were purchased from Proteintech, Inc (Wuhan, China), including anti-RARβ antibody (1:1,000, 14013-1-AP), anti-RXRA antibody (1:1,000, 83796-5-RR), anti-CRABP2 antibody (1:2000, 10225-1-AP), HRP-conjugated Goat Anti-Mouse IgG (1:4,000, SA00001-1). Anti-TRβ antibody (1:1,000, AF8157) and anti-BAX antibody (1:1,000, AF5120) were purchased form Beyotime, Inc (Shanghai, China). Anti-PPIB (1:1,000, 43603 S) and goat anti-rabbit IgG H&L (HRP) (1:10,000, ab205718) were products of Cell Signaling Technology, Inc (Shanghai, China) and Abcam (Cambridge, United Kingdom), respectively.

### 2.17 Methylation analysis via bisulfite sequencing PCR

A bisulfite sequencing PCR assay was conducted to analyze the methylation status of the RARB gene at promoter CpG sites across experimental samples. RL95-2 and THRB^(−/−)^/RL95-2 cells were treated with DMSO, 30 μM MPA, 10 and 30 μM CANA, as well as 10 μM MPA combined 30 μM CANA, and 30 μM MPA combined 30 μM CANA, respectively and each sample contained 1 × 10^6^ cells. Genomic DNA was extracted using the High-Efficiency Genomic DNA Extraction Kit (Tsingke Biotechnology Co., Ltd., Shanghai, China), and its concentration was quantified to ensure OD_260_/OD_280_ ratio within 1.8–2.0 and a concentration of 200–600 ng/μL. Bisulfite modification of DNA was performed using the DNA Bisulfite Conversion Kit (TianGen Biotech Co., Ltd., Beijing, China) with thermal cycling conditions of 95°C for 10 min, 64°C for 30–60 min, followed by a hold at 4°C, and purification according to the manufacturer’s protocol. The amplified products were purified, cloned into TA vectors using the E-pTO-T Quick Cloning Kit (General Biosystems Co., Ltd., Beijing, China), and transformed into *E. coli* for sequencing. The services of purifying and sequencing were offered by Hunan Platze Biotechnology Co., Ltd. Methylation analysis was performed using sequencing chromatograms, with methylated CpG sites represented by black circles and unmethylated CpG sites by white circles.

### 2.18 Statistical analysis

The data from three independent experiments are presented as the mean ± standard error of the mean (SEM). All statistical analyses were performed using Prism 9.5.2 (GraphPad Software, San Diego, CA, United States). Multiple group comparisons were conducted using one-way ANOVA (and nonparametric), followed by Tukey’s or Dunnett’s multiple comparison tests. Tukey’s *post hoc* test was conducted to make comparisons between any two groups throughout the experiment, while Dunnett’s test was used to make comparisons between the experimental and control groups. Two-tailed unpaired t-test was utilized to compare the transcriptomics prior to and after THRB knockout, the expression of RARβ within patients’ tissues and relative fluorescence intensity in Luciferase Assay. The statistical significance of the inhibition rate at various times was determined by repeated measurement. Data were considered statistically significant at *p*-values less than 0.05.

## 3 Results

### 3.1 Screening of selective candidates targeted TRβ

**FIGURE 1:**
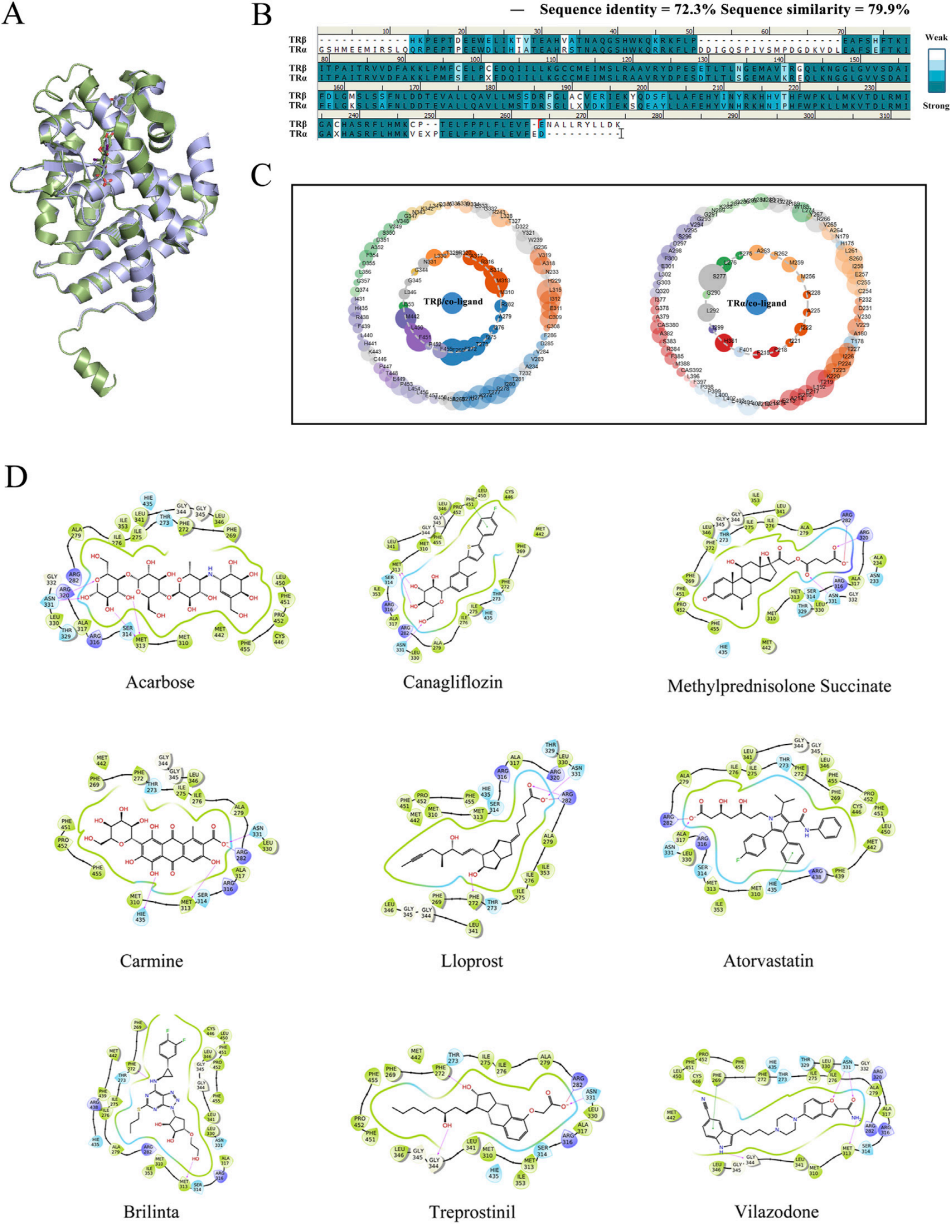
Virtual screening of selective candidates interacted with TRβ. **(A)** The 3D protein structures of TRα (green) and TRβ (blue). The green stick indicated the co-crystalline ligand of TRα, and the blue stick represented the co-crystalline ligand of TRβ. **(B)** One-dimensional amino sequence alignment of TRα and TRβ proteins. Identical residues were shown as dark blue, while similar residues were represented in light blue. **(C)** Asteroid plots generated by the Protein Contact Atlas. It visually demonstrated the relationship between protein and co-crystalline ligand. The innermost layer illustrated the critical amino acid residues and ions in direct contact with the co-crystallized molecule, while the outermost layer showed amino acid residues with indirect interactions. The circumference of each circle was proportional to the significance of the residue’s role, and coloring indicates the secondary structure of the residue. **(D)** Virtual screening candidate interaction with TRβ. The cartoons illustrated the top nine compounds achieved the best Docking score with TRβ. Pink arrows indicated hydrogen bonding interactions and green straight lines indicated π-π interactions. The number and strength of hydrogen and π-π interaction bonds help stabilize the complexes formed by proteins and ligands.

First of all, virtual screening was performed to identify a candidate that specifically targets TRβ. The structure differences between TRβ and TRα were compared, and the sequences as well as crystallographic structures of TRβ were superimposed using Discovery Studio 3.0 for analysis. Except for a few loop regions, the α-helix and β-sheet structures of both proteins aligned well, demonstrating their high homology (Figure 1A). TRα and TRβ exhibited a high degree of similarity, with a sequence identity of 72.3% and a sequence similarity of 79.9% (Figure 1B). Protein contact atlas analysis was conducted to explore the binding modes of crucial protein residues between TRβ/α and their co-crystalline ligands for molecular binding. The results showed that the amino acid MET313, PHE451 and PHE269 were critical for TRβ to interact with the co-ligand, while the amino acid sites SER277 and HIS381 were the primary residues involved in the interaction with TRα (Figure 1C). The results defined the principles for the following virtual screening.

In the process of virtual screening, the modes of action of the candidate compounds interacting with TRβ were analyzed using the Glide suite of Schrödinger software, generating 975 complexes that interacted with at least one of the amino acids in MET313, PHE451, ASN331 or PHE269 but not SER277 and HIS381, which ensured the specific combination with TRβ. The 975 candidate complexes were then ranked in ascending order in terms of their Docking score. The binding modes of the top nine compounds were shown in Figure 1D, with the best Docking score listed in Table 1. Based on the Docking scores, XP Gscore, Glide Gscore, and MM GBSA ∆G _Bind,_ along with the mode of action of the complexes, acarbose and CANA were ultimately selected to measure cell viability due to their highest Docking scores, and atorvastatin was included as well since it demonstrated a strong binding affinity to TRβ with an excellent molecular docking score value of −14.294 kcal mol^-1^.

**TABLE 1:** Virtual screening of drug candidate docking rankings.

Name	Docking score (kcal mol^-1^)	XP gScore (kcal mol^-1^)	Glide gscore (kcal mol^-1^)	MM GBSA ∆G _Bind_ (kcal mol^-1^)
Acarbose	−17.039	−17.521	−17.521	−65.03
Canagliflozin	−15.224	−15.225	−15.225	−43.42
Methylprednisolone Succinate	−14.661	−14.661	−14.661	−59.1
Carmine	−14.569	−15.664	−15.664	−86.46
Lloprost	−14.503	−14.503	−14.503	−48.98
Atorvastatin	−14.294	−14.296	−14.296	−2.66
Brilinta	−13.823	−13.824	−13.824	−74.78
Treprostinil	−13.664	−13.664	−13.664	−36.01
Vilazodone	−13.363	−13.498	−13.498	−76.88

**FIGURE 2:**
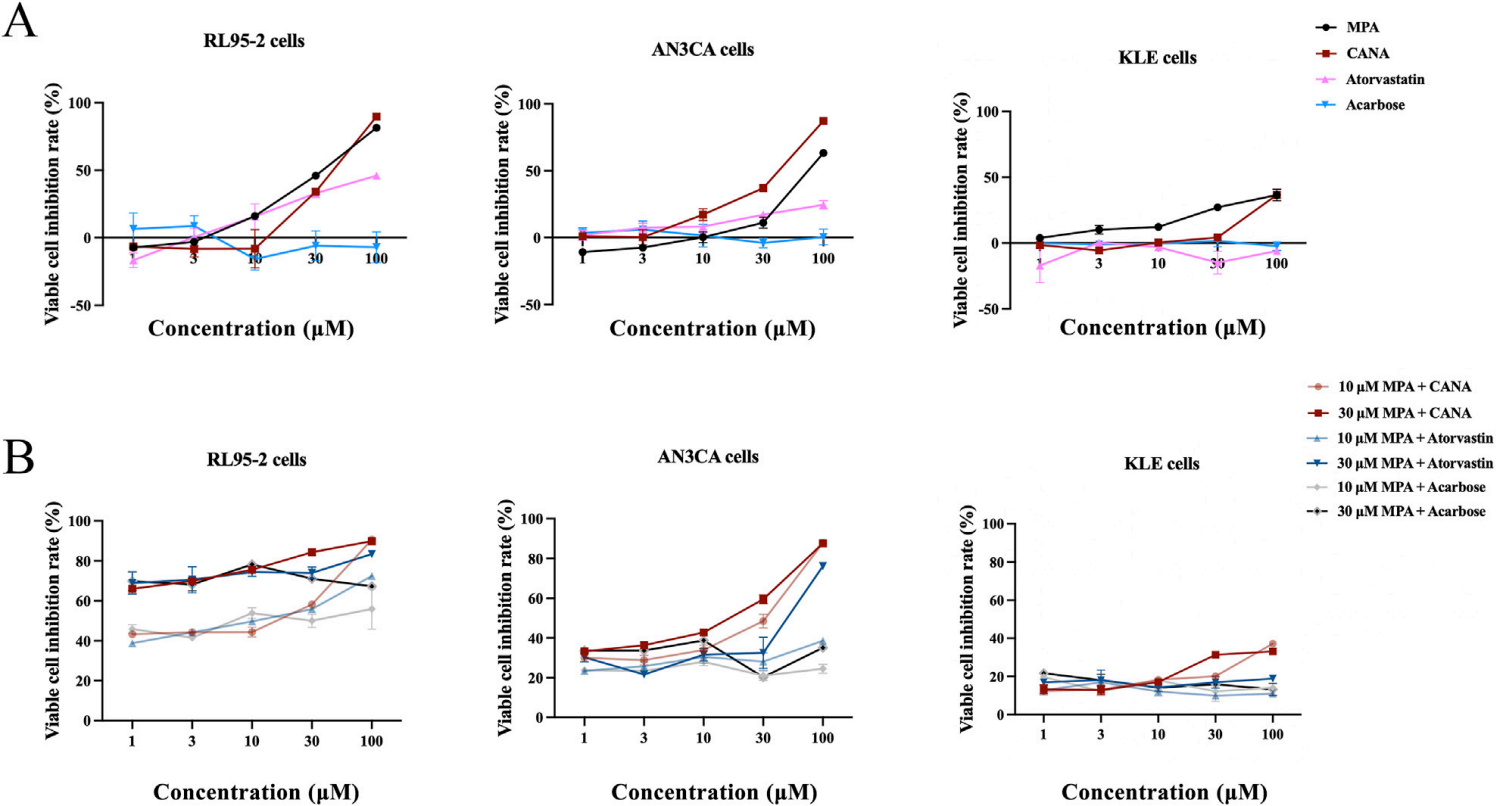
Inhibitory effect of MPA, CANA, atorvastatin and acarbose on RL95-2、AN3CA and KLE cells. **(A)** Inhibitory effect of MPA, CANA, atorvastatin and acarbose on RL95-2, AN3CA and KLE cells. **(B)** Inhibitory effect of CANA, atorvastatin and acarbose in combination with 10 or 30 μM MPA on RL95-2, AN3CA and KLE cells. Data were calculated from triplicate wells in three independent assays.

After virtual screening, the effects of MPA, CANA, atorvastatin and acarbose were assessed on type I and II EC cells at the concentration range from 1 to 100 μM. Both MPA and CANA inhibited the viability of RL95-2, AN3CA and KLE cells in a concentration-dependent manner. Compared with MPA, CANA demonstrated stronger inhibitory effects on AN3CA and KLE cells with the lowest IC_50_ value but had a similar effect on RL95-2 cells (Figure 2A). In contrast, acarbose and atorvastatin exhibited weak or negligible effects on the viability of the 3 cell lines. When combined with 10 μM MPA, CANA exhibited the strongest inhibitory effects on the 3 cells with the lowest IC_50_ value compared to acarbose and atorvastatin. Similarly, CANA exhibited the strongest inhibitory effects among the three candidate compounds on the AN3CA and KLE cells with the lowest IC_50_ value when it was combined with 30 μM MPA, except that atorvastatin combined with 30 μM MPA displayed the strongest inhibitory activities on RL95-2 cells. The results were shown in Figure 2B and Table 2.

**TABLE 2:** Antiproliferative activity of various compounds on three types of human endometrial cell lines after treatment for 48 h, as presented as IC_50_ (95% confidence interval) values (μM).

Compounds		Inhibitory potency IC_50_ (95% CI) μM		
		RL95-2 cells	AN3CA cells	KLE cells
MPA		34.15 (22.80–52.79)	77.96 (48.73–141.9)	260.0 (132.0–1,057)
CANA		39.28 (28.31–55.24)	36.89 (25.98–52.23)	152.1 (98.81–488.6)
Atorvastatin		100.2 (34.49–16,724)	—	—
Acarbose		—	—	—
CANA	10 μM MPA	5.260 (/–/)	16.06 (0.37–54.86)	659.2 (140.1 – ∞ )
	30 μM MPA	0.1481 (0.01–0.47)	8.89 (1.15–43.28)	733.8 (142.4 - ∞ )
Atorvastatin	10 μM MPA	7.14 (2.25–17.97)	—	—
	30 μM MPA	0.01 (0.001–0.18)	35.69 (/-/)	—
Acarbose	10 μM MPA	18.50 (/-/)	—	—
	30 μM MPA	—	—	—

Data were calculated from triplicate wells in three independent assays and presented as IC_50_ (95% confidence interval) values (μM)./meant that the corresponding IC_50_ value was greater than 800 μM or could not be calculated because the viability of cells did not reach 50% of the maximum.

### 3.2 Establishment of THRB^(−/−)^/RL95-2 EC cells and detection of resistance to MPA

To confirm the roles of TRβ in the growth of EC cells and progestin resistance, THRB knockout (KO) RL95-2 cells, named as THRB^(−/−)^/RL95-2 cells were constructed.

**FIGURE 3:**
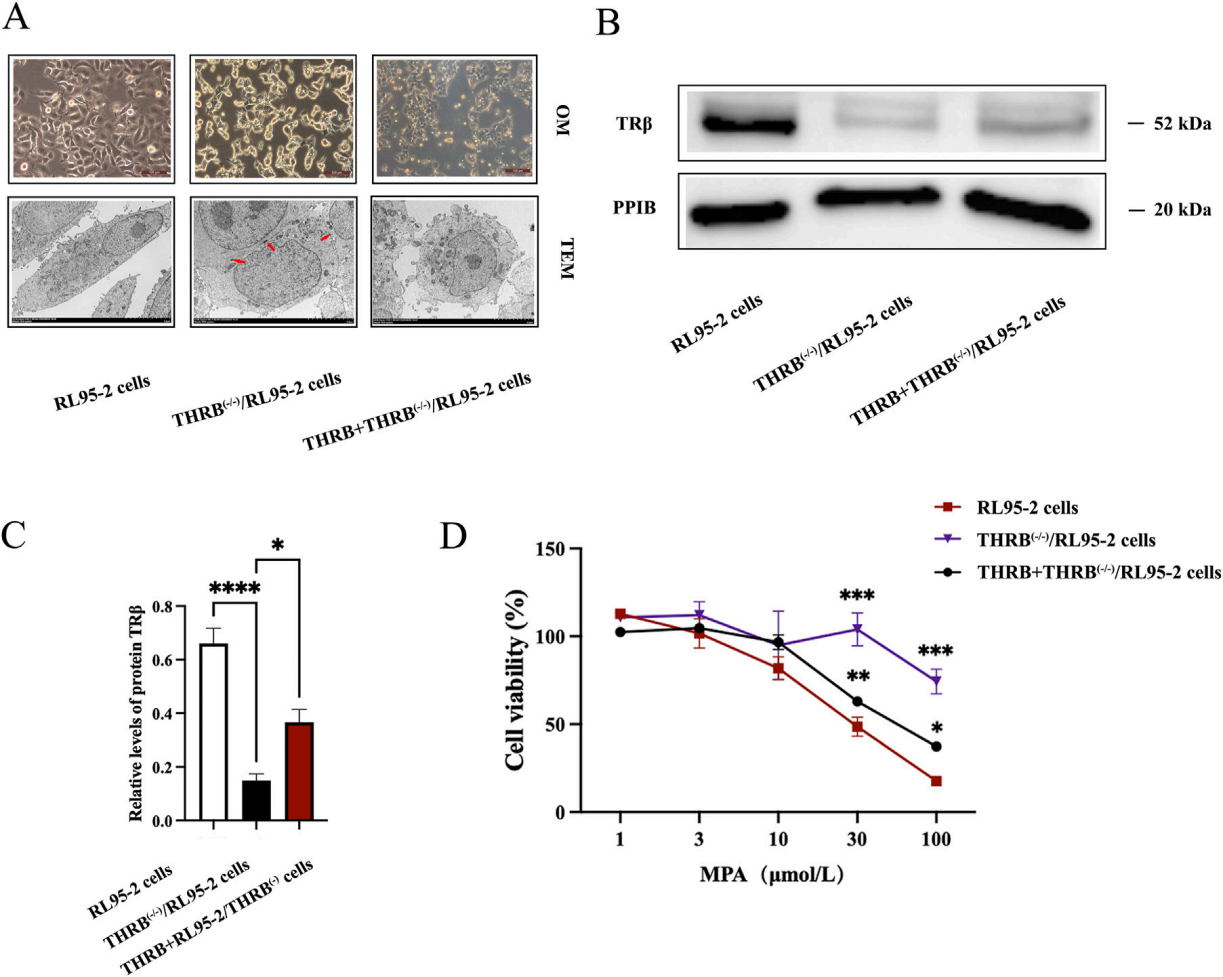
Changes in morphology and sensitivity to MPA in THRB^(−/−)^/RL95-2 cells. **(A)** Morphology of RL95-2, THRB^(−/−)^/RL95-2 and THRB + THRB^(−/−)^/RL95-2 cells under optical microscopy (OM) and transmission electron microscopy (TEM). **(B,C)** THRB^(−/−)^/RL95-2 cells knockout efficiency and THRB + THRB^(−/−)^/RL95-2 cells backfill efficiency. **(D)** Effects of MPA on viability of RL95-2, THRB^(−/−)^/RL95-2 and THRB + THRB^(−/−)^/RL95-2 cells. The results were presented as the mean ± SEM from three independent experiments with triplet repeat of each data. *p < 0.05, **p < 0.01,***p < 0.001, ****p < 0.0001 compared with RL95-2 cells.

Unlike the monolayer adherent growth of wild-type RL95-2 cells, THRB^(−/−)^/RL95-2 cells exhibited a significant morphological change, characterized by a noticeable decrease in cell size, a spherical shape, and clustering in piles. After reinducing THRB to the THRB^(−/−)^/RL95-2 cells, the cell morphology reverted, and some of them grew in a monolayer with an adherent layer again (Figure 3A; Supplementary Figure S1) Indeed, in addition to changes in cell morphology, the mode of communication in THRB^(−/−)^/RL95-2 cells did change from gap junction to tight junctions based on ultrastructure like desmosomes, which are marked by red arrow. TRβ was completely knocked out in THRB^(−/−)^/RL95-2 cells (*p* < 0.001), while reinducing THRB partially restored the expression of TRβ (*p* < 0.05; Figures 3B,C; Supplementary Figure S2). After treatment with 30 and 100 μM MPA, THRB^(−/−)^/RL95-2 cells showed significantly higher viability than RL95-2 cells (*p* < 0.001), indicating resistance to MPA suppression (Figure 3D). These results indicated that THRB^(−/−)^/RL95-2 cells displayed distinct morphology from the wild-type cells and a unique connection, exhibiting resistance to MPA, which was partially reversed by TRβ reinduction.

### 3.3 CANA combined MPA suppressed proliferation and promoted apoptosis in both RL95-2 and THRB^(−/−)^/RL95-2EC cells

**FIGURE 4:**
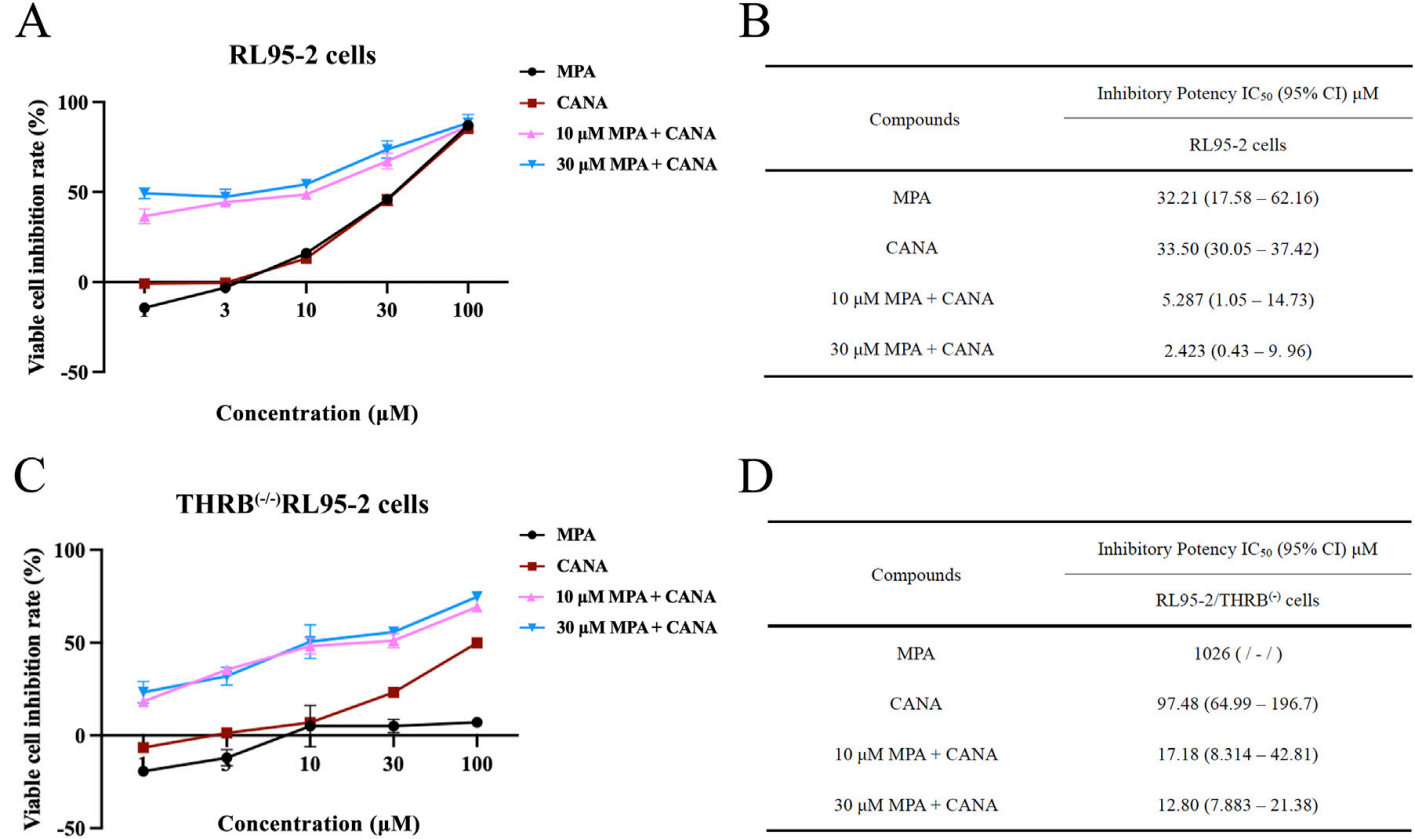
The effect of MPA, CANA and their combination on the viability and inhibition rates of RL95-2 and THRB^(−/−)^/RL95-2 cells. **(A,B)** Effects of MPA, CANA, and their combination on the viability and inhibition rates of RL95-2 cells after treatment for 48 h. **(C,D)** Antiproliferation activity of MPA, CANA, 10 μM MPA combined CANA and 30 μM MPA combined CANA on THRB^(−/−)^/RL95-2 cells after treatment for 48 h, as presented as IC_50_ (95% confidence interval) values (μM). The results were presented as IC_50_ (inhibits cell proliferation by 50%) and its 95% CI (confidence interval). IC_50_ was defined as the drug concentration required to reduce the number of living cells by 50% after incubation with 1–100 μM of the compounds. Data were calculated from triplicate wells in three independent assays and presented as IC_50_ (95% confidence interval) values (μM)./meant that the corresponding IC_50_ value was greater than 800 μM or could not be calculated because the viability of cells did not reach 50% of the maximum.

The inhibitory effects of MPA, CANA and their combination on the viability of two types of EC cell lines, RL95-2 and THRB^(−/−)^/RL95-2 cells were evaluated. For RL95-2 cells, the treatment with MPA and CANA at concentrations ranging from 1 to 100 μM and with combinations of 10 or 30 μM MPA and CANA inhibited the viability of RL95-2 cells in a concentration-dependent manner. The impedance rates reached the maximum when the cells were treated with 30 μM MPA plus 1–100 μM CANA, yielding an IC_50_ (95% CI) value of 2.423 (0.43–9.96) (Figures 4A,B).

In THRB^(−/−)^/RL95-2 cells, MPA did not suppress the proliferation of the cells, but CANA remained to inhibit the cell proliferation in a concentration-dependent manner, with an IC_50_ (95% CI) value was 97.48 (64.99–196.7). When 30 μM MPA was combined with 1–100 μM CANA, the inhibition rate reached its maximum, and the IC_50_ (95% CI) value was 12.80 (7.883–21.38) (Figures 4C,D). There was no remarkable difference in the IC_50_ (95% CI) values between 10 and 30 μM MPA in combination with CANA, respectively.

**FIGURE 5:**
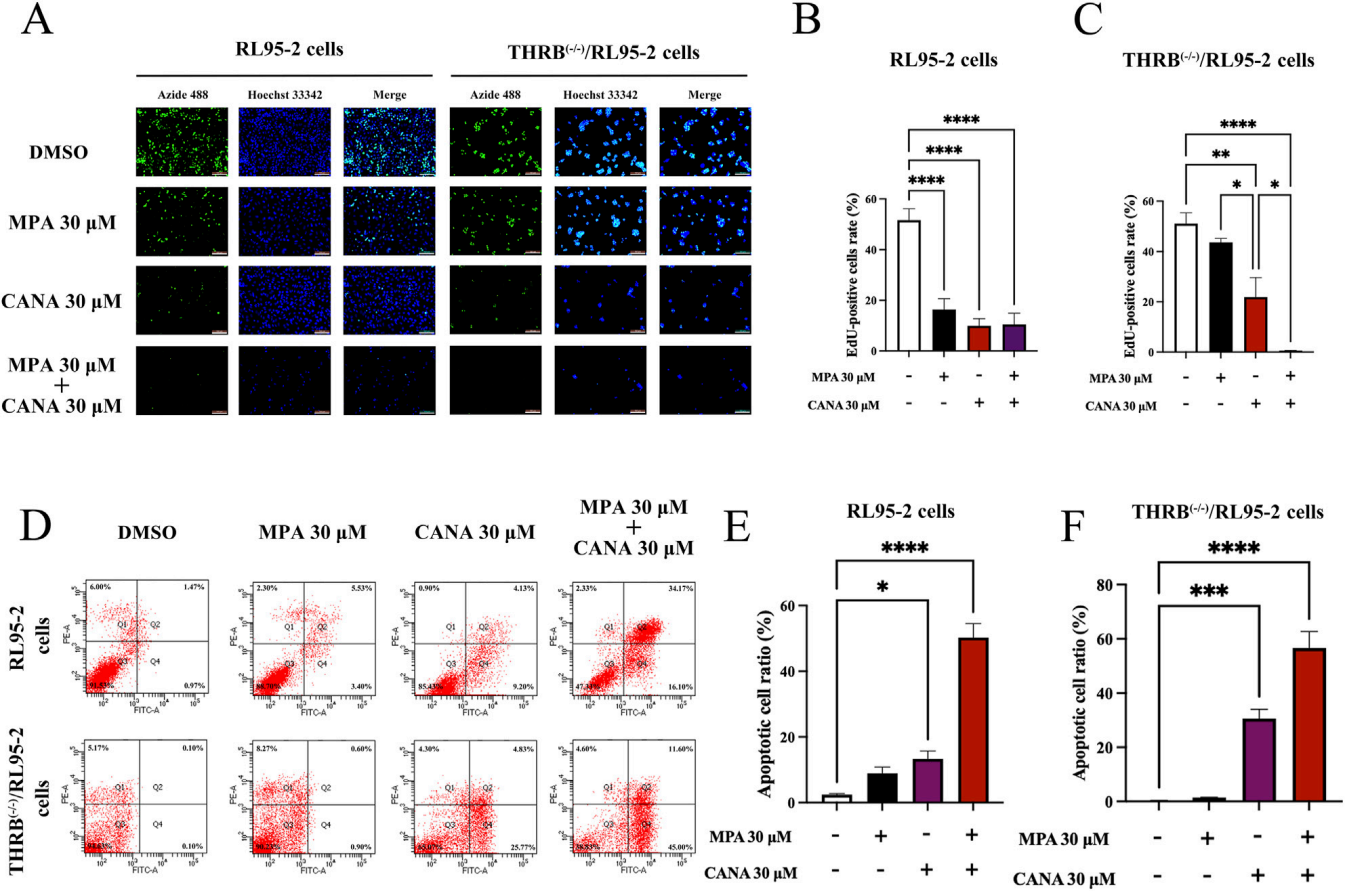
Changes of proliferation, apoptosis and migration ability in RL95-2 and THRB^(−/−)^/RL95-2 cells before and after 30 μM CANA and 30 μM MPA treatment for 48 h. **(A–C)** The changes of cell proliferation and EdU-positive cells ratio. All cell nuclei showed blue fluorescence indicative of Hoechst 33,342 staining. EdU-labeled cells suggested new DNA synthesis. **(D–F)** The changes of cell apoptosis capacity and percentage of apoptotic cells. The results were presented as the mean ± SEM from three independent experiments with triplet repeat of each data. *p < 0.05, **p < 0.01, ***p < 0.001, ****p < 0.0001 compared with the control group.

Moreover, the EdU-incorporation assay was conducted to measure the proliferation of RL95-2 and THRB^(−/−)^/RL95-2 cells. In RL95-2 cells, the positive ratio of EdU staining decreased significantly from 51.04% ± 10.04% in the control cells to 16.37% ± 9.57% (*p* < 0.001), 9.90% ± 6.28% (*p* < 0.001), and 10.41% ± 9.99% (*p* < 0.001) after treatment with MPA, CANA, and their combination at the concentration of 30 μM for 48 h, respectively (Figures 5A,B). In THRB^(−/−)^/RL95-2 cells, no significant difference was observed between the control (51.02% ± 9.69%) and the MPA-treated cells (43.65% ± 3.47%, *p* > 0.05), as shown in Figure 5C; Supplementary Figures S3, S4. However, CANA alone or in combination with MPA treatment significantly reduced the EdU staining rate from 51.02% ± 9.69% in the control group to 21.91% ± 11.29% (*p* = 0.002) and 0.57% ± 0.32% (*p* < 0.001), respectively. Notably, 30 μM CANA decreased the proliferation rate of the cells more effectively than MPA (*p* = 0.02).

Flow cytometry was used to further assess apoptosis of RL95-2 and THRB^(−/−)^/RL95-2 cells. In RL95-2 cells, the apoptosis rate increased from 2.43% ± 0.49% in the control group to 8.93% ± 3.27% (*p* > 0.05), 13.33% ± 4.16% (*p* < 0.05), and 50.27% ± 7.41% (*p* < 0.001) after treatment with MPA, CANA, and their combination at the concentration of 30 μM for 48 h, respectively (Figures 5D,E). In THRB^(−/−)^/RL95-2 cells, the apoptosis rate rose from 0.20% ± 0.35% in the control cells to 1.50% ± 0.10% (*p* > 0.05), 30.60% ± 5.99% (*p* < 0.001), and 56.60% ± 10.51% (*p* < 0.001), respectively following the same treatments (Figure 5F). The results demonstrated that MPA effectively inhibited proliferation and promoted apoptosis in RL95-2 cells, but not in THRB^(−/−)^/RL95-2 cells. In contrast, CANA exhibited these effects in both RL95-2 and THRB^(−/−)^/RL95-2 cells.

### 3.4 CANA combined with MPA reduced the growth of xenograft tumors induced by both of RL95-2 and THRB^(−/−)^/RL95-2EC cells in nude mice

**FIGURE 6:**
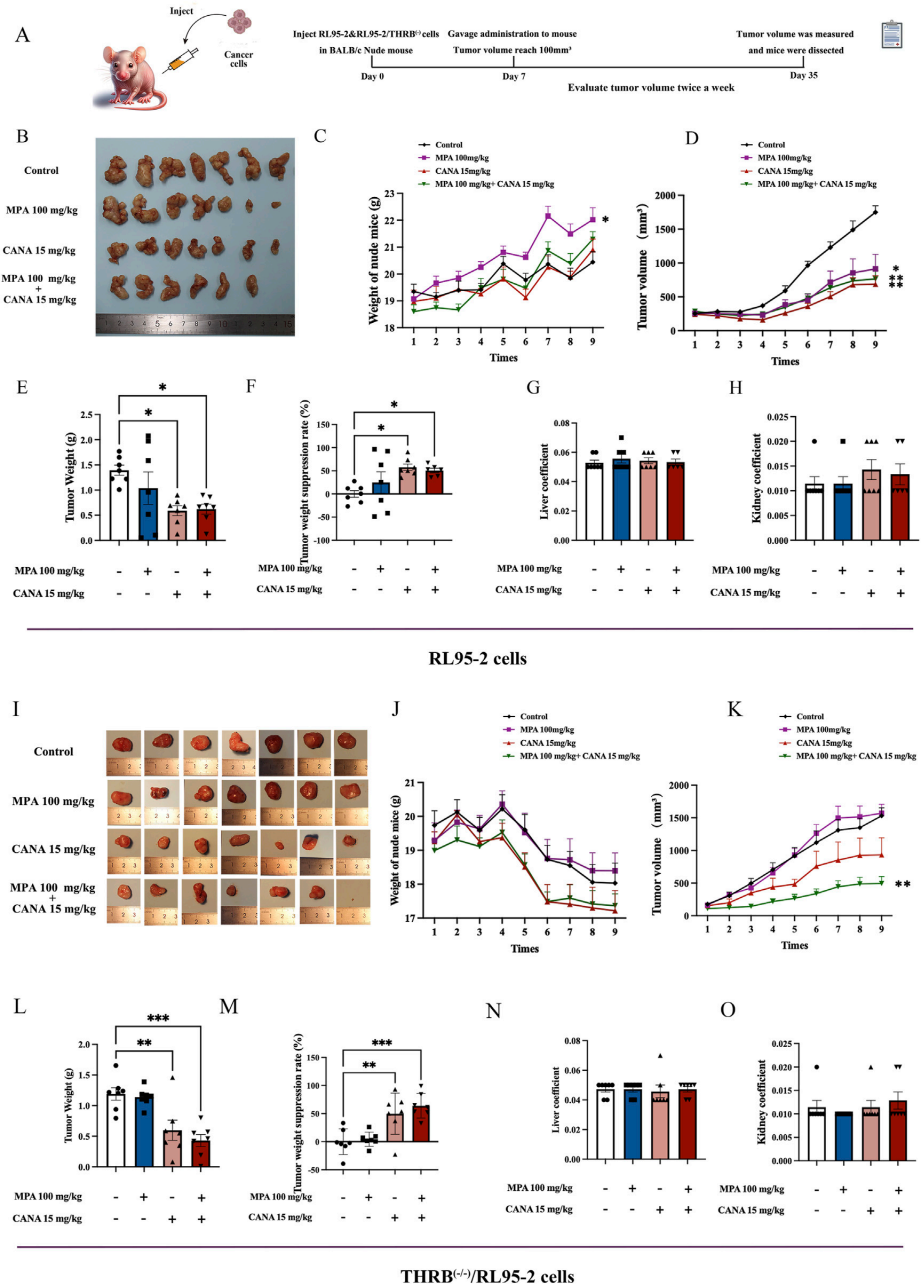
Effects of MPA, CANA and their combination on the growth of transplanted tumors in nude mice, respectively after 28 days of administering 100 mg/kg MPA and 15 kg/kg CANA and their combination (n = 7). **(A)** Experimental protocols for establishing xenograft tumor in nude mice and treatment. **(B)** Tumor shape of the xenograft tumors induced by RL95-2 cells. **(C)** The changes of body weights in the nude mice with xenograft tumors induced by RL95-2 cells. **(D)** Tumor sizes of the nude mice with xenograft tumors induced by RL95-2 cells. **(E,F)** Xenograft tumor weights and tumor weight suppression rates in the nude mice inoculated with RL95-2 cells. **(G,H)** Liver and kidney coefficient of the nude mice with xenograft tumor induced by RL95-2 cells. **(I)** Tumor shape of the xenograft tumors induced by THRB^(−/−)^/RL95-2 cells. **(J)** The changes of body weights of the nude mice with xenograft tumors induced by THRB^(−/−)^/RL95-2 cells. **(K)** Tumor volume of the nude mice with xenograft tumor induced by THRB^(−/−)^/RL95-2 cells. **(L,M)** Xenograft tumor weights and tumor weight suppression rates in the nude mice inoculated with THRB^(−/−)^/RL95-2 cells. **(N,O)** Liver and kidney coefficient of the nude mice with xenograft tumors induced by THRB^(−/−)^/RL95-2 cells. *p < 0.05, **p < 0.01, ***p < 0.001, ****p < 0.0001 compared with the control group.

Two types of xenograft tumors induced by RL95-2 cells and THRB^(−/−)^/RL95-2 cells were established to evaluate the inhibitory effects of CANA and its combination with MPA on proliferative activity *in vivo*. Before treatment, the average tumor size was around 100 mm^3^ in all groups, and no remarkable differences among all groups (*p* > 0.05) (Figure 6A).

In the mice inoculated with RL95-2 cells, the gross morphology of the xenografts displayed an irregular cauliflower shape (Figure 6B). The mice maintained stable body weight throughout the experiment. The 100 mg/kg MPA-treated mice exhibited a significant increase in body weight compared to that of the control group (*p* = 0.021; Figure 6C). After 28 days of treatment, the mean tumor weight reached 1.39 ± 0.27 g in the control group. Significant reductions in tumor volumes were observed in the 15 mg/kg CANA, and 15 mg/kg CANA combined 100 mg/kg MPA treatment groups, with tumor weights of 0.59 ± 0.26 g (p < 0.05) and 0.70 ± 0.17 g (p < 0.05), respectively (Figure 6D). In addition, 100 mg/kg MPA treatment alone failed to significantly reduce the tumor weight compared to the control group (*p* > 0.05; Figures 6E,F), while the treatments of 100 mg/kg MPA, 15 mg/kg CANA, and their combination were effective in inhibiting the volume increase of tumor sizes (*p* < 0.01). After 28 days of treatment, no remarkable differences were observed in the liver and kidney coefficients between the groups of treatment and control (Figures 6G,H). During the experiment, one nude mouse died during gavage in the group of MPA and CANA combination treatment.

In the mice inoculated with THRB^(−/−)^/RL95-2 cells, the gross morphology of the xenografts appeared more regularly round, with some of them containing pus (Figure 6I). The body weights of all mice experienced a steady decrease from the second week being inoculated with the cells subcutaneously (Figure 6J). MPA treatment alone did not significantly reduce tumor sizes compared to the control group. However, 15 mg/kg CANA and the combination with MPA significantly reduced the tumor sizes compared to the control mice (*p* < 0.01) (Figure 6K). The mean tumor weights reached 1.19 ± 0.27 and 1.14 ± 0.15 g in the control and 100 mg/kg MPA treatment mice, respectively, and no difference was found between the two groups (*p* > 0.05). In contrast, the tumor weights in the groups of 15 mg/kg CANA and CANA-combined MPA (15 mg/kg plus 100 mg/kg) treatment were 0.60 ± 0.44 g (*p* = 0.003) and 0.43 ± 0.26 g (*p* < 0.0001), respectively (Figures 6L,M). After 28 days of treatment, no pronounced differences were observed in the liver and kidney coefficients of the treatment groups compared to those of the control groups (Figures 6N,O). These results indicated that CANA alone and in combination with MPA had anti-tumor effects in mice.

### 3.5 Combined transcriptomic and proteomic revealed potential target genes for MPA resistance in EC due to TRβ deletion

Furthermore, transcriptomic and proteomic analysis were utilized to explore the potential target genes associated with MPA resistance in the THRB-knockout RL95-2 cells.

**FIGURE 7:**
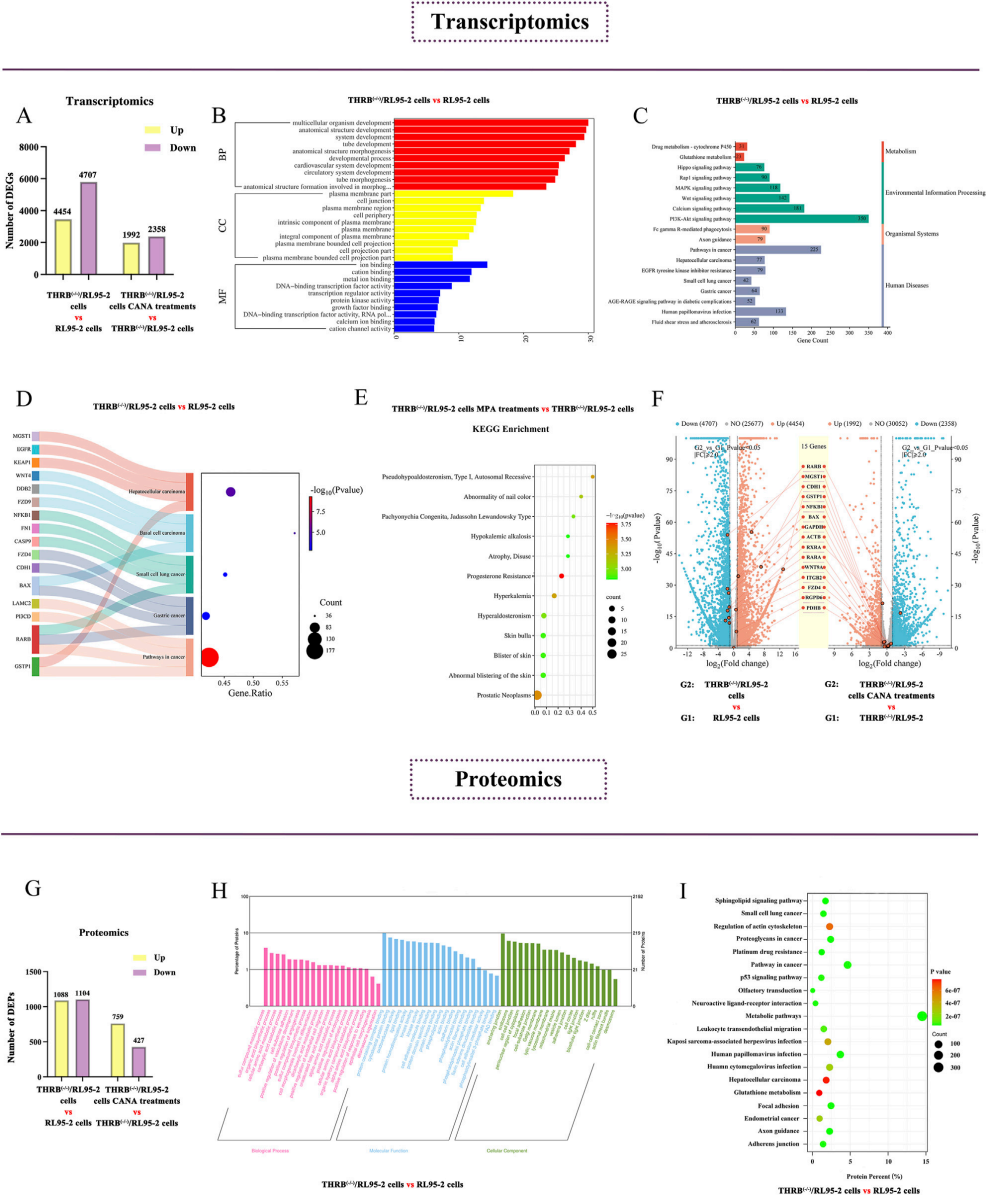
Screening key DEGs and DEPs in the progestin-resistant THRB^(−/−)^/RL95-2 EC cells via transcriptomic and proteomic assay. **(A)** Column chart showing the numbers of DEGs up and downregulated in THRB^(−/−)^/RL95-2 cells versus RL95-2 cells via transcriptomics. **(B)** GO enrichment analysis of DEGs of THRB^(−/−)^/RL95-2 versus RL95-2 cells in transcriptomics. **(C)** KEGG enrichment analysis of DEGs of THRB^(−/−)^/RL95-2 versus RL95-2 cells in transcriptomics. **(D)** KEGG enrichment of tumor-related signaling pathways in transcriptomics of THRB^(−/−)^/RL95-2 versus RL95-2 cells. **(E)** Disease enrichment analysis of THRB^(−/−)^/RL95-2 cells in transcriptomics before and after MPA administration. **(F)** DEGs within THRB^(−/−)^/RL95-2 versus RL95-2 and CANA 30 μM treatment THRB^(−/−)^/RL95-2 cells versus the control cells, shown as double volcano plots. The transcriptomic results were represented as mean ± SEM of three independent experiments. **(G)** Differentially expressed protein of THRB^(−/−)^/RL95-2 versus RL95-2 cells in proteomics. **(H)** GO enrichment analysis of THRB^(−/−)^/RL95-2 versus RL95-2 cells in proteomics before and after DMSO treatment. **(I)** KEGG enrichment analysis of THRB^(−/−)^/RL95-2 versus RL95-2 cells in proteomics before and after DMSO administration.

In the transcriptomic analysis, there were 9,161 DEGs identified in the THRB^(−/−)^/RL95-2 cells compared with that in RL95-2 cells, including 4,454 upregulated and 4,707 downregulated DEGs (Figure 7A). GO enrichment analysis revealed significant alterations in the plasma membrane, cellular junction functions, and transcription factor activities before and after THRB knockout (Figure 7B). In KEGG enrichment analysis, the more significant entries were categorized according to metabolism, environmental information processing, organismal systems, and human diseases, including several cancer-related pathways, such as hepatocellular carcinoma, small-cell lung cancer, and gastric cancer (Figure 7C). Thereinto, RARB was found to be involved in multiple cancer-related pathways (Figure 7D). Furthermore, disease enrichment analysis exhibited that MPA-resistance was significantly elevated in THRB^(−/−)^/RL95-2 cells but not in wild-type RL95-2 cells (Figure 7E).

After administration of 30 μM CANA, 4,350 DEGs were identified compared with that of DMSO treatment in the THRB^(−/−)^/RL95-2 cells, with 1,992 upregulated and 2,358 downregulated DEGs. Dual-volcano plot analysis demonstrated that the expression of RARβ was significantly elevated in THRB^(−/−)^/RL95-2 cells compared to that of RL95-2 cells, and with a pronounced reduction after CANA treatment (Figure 7F).

Proteomic analysis identified 2,192 DEPs in THRB^(−/−)^/RL95-2 cells compared to RL95-2 cells, including 1,088 upregulated and 1,104 downregulated DEPs (Figure 7G). GO enrichment analysis displayed significant alterations in the cellular junction functions and amino acid metabolism before and after THRB knockout in RL95-2 cells (Figure 7H). KEGG enrichment analysis demonstrated hepatocellular carcinoma and glutathione metabolism signaling pathway were significantly changed (Figure 7I). Following CANA treatment, THRB^(−/−)^/RL95-2 cells exhibited 1,186 DEPs compared to those treated with DMSO, involving 759 upregulated and 427 downregulated DEPs. Specifically, the signaling pathways of retinoic acid were identified *via* proteomics, including cellular retinoic acid-binding Protein 2 (CRABP2) and Retinoid X Receptor α (RXRA) which exhibited significant upregulation in THRB^(−/−)^/RL95-2 cells following THRB knockout. After treatment with 30 μM CANA, the expression of these proteins was markedly reduced, indicating CANA could modulate the expression of proteins involved in retinoic acid signaling in THRB^(−/−)^/RL95-2 cells (Table 3). Transcriptomics and proteomics as a whole identified key DEPs of the THRB^(−/−)^/RL95-2 cells, including cellular junctions, metabolism, and cancer-related pathways, suggesting RARβ, CRABP2, and RXRA could be potential targets for MPA resistance and targeted by CANA.

**TABLE 3:** Retinoic acid-related protein expression in proteomics.

Gene names	DMSO treatment protein expression quantity in RL95-2 cells	DMSO treatment protein expression quantity in THRB^(−/−)^/RL95-2 cells	CANA treatment protein expression quantity in THRB^(−/−)^/RL95-2 cells
CRABP2	120.11	7,936.66	2,400.89
RBP1	2,313.94	2,124.41	3,344.71
RBP4	49.99	43.98	70.45
RDH10	276.20	93.99	81.35
RDH13	480.97	646.52	788.34
RXRA	110.55	324.44	220.61

### 3.6 Molecular dynamics simulations verified the tightly binding affinity between RARβ and CANA

**FIGURE 8:**
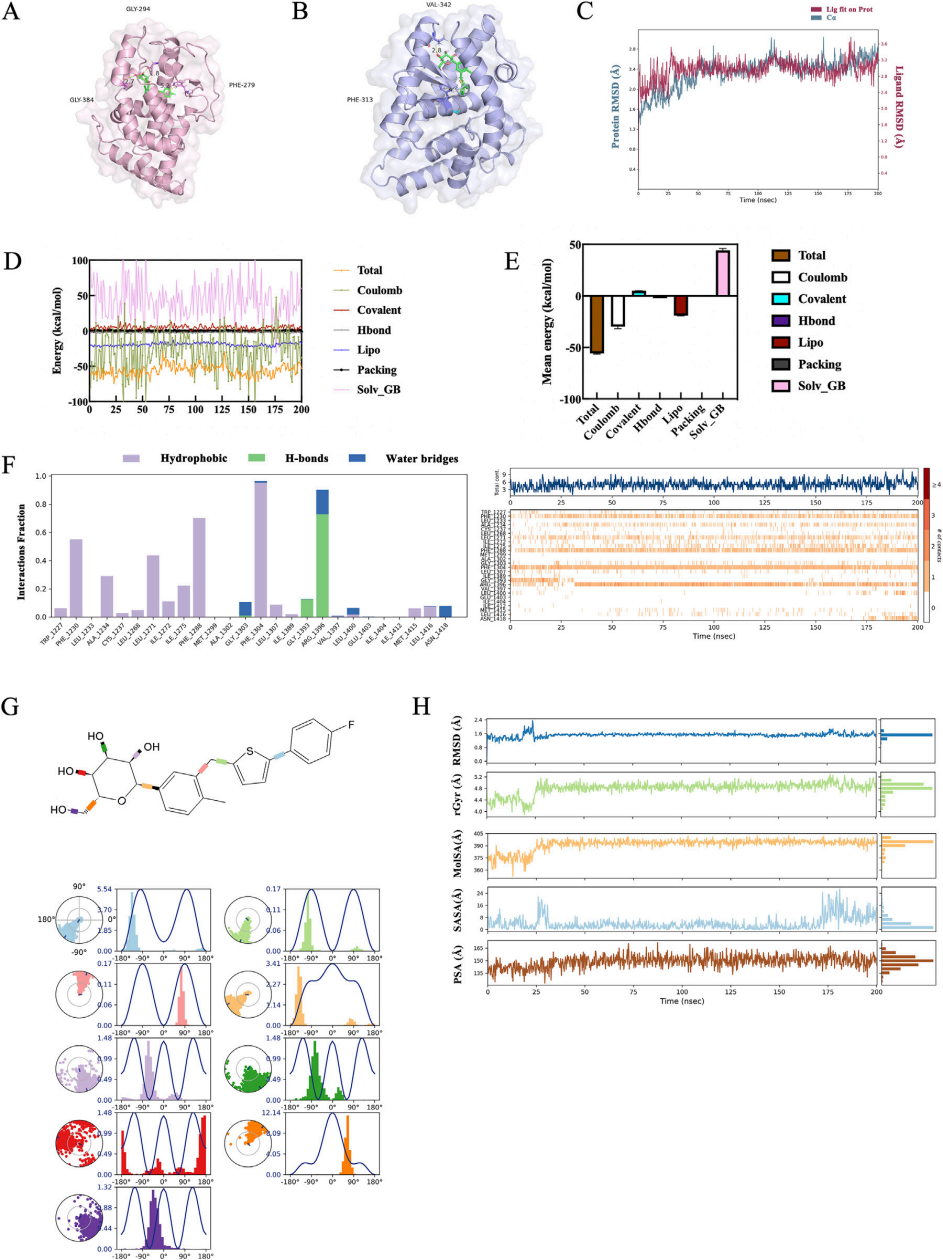
Molecular dynamics simulation of CANA/RARβ complex. **(A,B)** Simulation mode of molecular interactions of RARβ/CANA and RXRA/CANA assayed by molecular docking. In the figure, RARβ was depicted as a pink cartoon, RXRA as a blue cartoon, and CANA as a green stick representation. **(C)** RMSD curves of RARβ protein backbone atoms throughout the 200 ns molecular dynamics simulation. **(D)** The spectrum of binding free energies of RARβ/CANA complex determined by MM-GBSA calculation throughout the 200 ns simulation. **(E)** The binding free energy mean values of RARβ/CANA complex determined by MM-GBSA calculation throughout the 200 ns molecular dynamics simulation. **(F)** Molecular interactions of RARβ with CANA assayed by molecular dynamics simulation. **(G)** Statistical diagram of protein–ligand contacts between RARβ and CANA and CANA’s five properties obtained from molecular dynamics simulation over the entire 200 ns for complexes. Each CANA rotary key was represented by a different color, and the radar chart of the corresponding color indicated the rotation angle of each rotary key. **(H)** Ligand Torsion Profile obtained from molecular dynamics simulation over the entire 200 ns for complex.

Molecular docking analysis was conducted to evaluate the binding affinity between DEPs and CANA. CANA was examined for molecular docking with key proteins such as RARβ, CRABP2 and RXRA, respectively, but the results showed that CANA could only form stable binding interactions with RARβ and RXRA. The results also demonstrated that CANA interacted with RARβ *via* π-π interactions at the PHE1230 and PHE1288 residues, achieving a docking score of −13.886 kcal/mol. Similarly, CANA interacted with RXRA through π-π interactions at the PHE313 residue and a hydrogen bond at the VAL342 residue, yielding a docking score of −10.630 kcal/mol (Figures 8A,B). A 200 ns molecular dynamics simulation in a simulated aqueous environment was performed to evaluate conformational changes in the amino acid backbone and ligand. In the analysis of root mean square deviation (RMSD), the RARβ/CANA complex exhibited gradual stabilization after minor fluctuations during the first 30 ns, with values ranging from 2.0 to 2.7 Å (Figure 8C). Binding strength was quantified using the MM-GBSA method, which reliably correlated with experimental activity. The ΔG values were derived from 200 frames sampled across the 200 ns molecular dynamics simulation (Figure 8D), and the RARβ/CANA complex exhibited a ΔG_bind_total of −55.80 kcal/mol, showing excellent binding affinity results (Figure 8E).

The molecular dynamics simulation results demonstrated there was a consistent π-π interaction between CANA and the PHE1288 and PHE1304 of RARβ throughout the simulation. Additionally, the ARG1396 of RARβ formed a stable hydrogen bond with CANA’s hydroxyl group, which further stabilized the RARβ/CANA complex (Figure 8F).

The molecular dynamics simulations showed that the rotational bonds of CANA remained concentrated at fixed angles, indicating CANA conformational stability (Figure 8G). In addition, ligand properties, including RMSD, radius of gyration (rGyr), molecular surface area (MolSA), solvent-accessible surface area (SASA), and polar surface area (PSA), were further analyzed to evaluate the physicochemical properties and stability of CANA. The RMSD curve for CANA remained stable and average values were around 1.3 Å. After initial fluctuations in the first 30 ns, the rGyr, PSA, SASA and MolSA curves remained stable throughout the simulation, as indicated by nearly constant values and straight-line graphs, signifying a stable complex structure of RARβ and CANA (Figure 8H). These results demonstrated that RARβ was likely a potential target of CANA.

### 3.7 CANA treatment attenuated aberrant retinoic acid signaling activation in THRB^(−/−)^ RL95-2 cells

**FIGURE 9:**
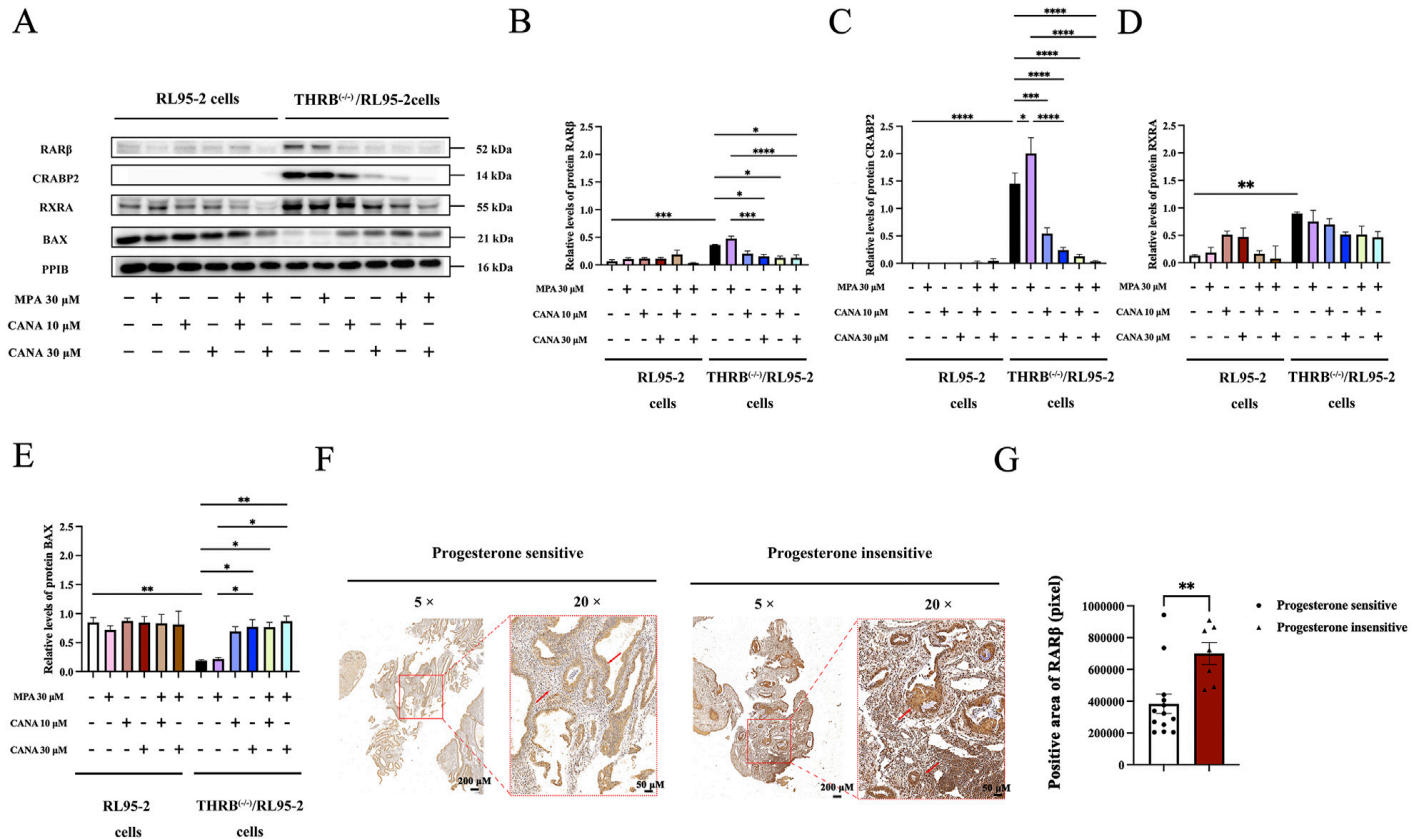
The expression of retinoic acid relevant proteins in THRB^(−/−)^/RL95-2 cells, validated by Western blot or immunohistochemical analysis. **(A–E)** The protein expression of RARβ, CRABP2, RXRA and BAX in RL95-2 and THRB^(−/−)^/RL95-2 cells treatment with MPA, CANA, and their combination. **(F,G)** RARβ immunohistochemical analysis and differential protein expression statistics of RARβ in progestin sensitive (n = 13) or insensitive uterine tissues (n = 7) (**P < 0.01). The results were presented as the mean ± SEM from three independent experiments with triplet repeats of each data. *p < 0.05, **p < 0.01, ***p < 0.001, ****p < 0.0001, comparation conducted between any two groups.

Based on the results of transcriptomics, proteomics, and molecular simulation, RARβ, CRABP2 and RXRA were screened out and further examined (Figure 9A; Supplementary Figure S5). Knocking out THRB significantly elevated the protein levels of RARβ and CRABP2 compared to those in wild-type RL95-2 cells, with RARβ increasing by 5.73-fold and CRABP2 by 145-fold (*p* < 0.001). In the THRB^(−/−)^/RL95-2 cells, treatment with 30 μM MPA did not remarkably alter the protein expression of RARβ but enhanced the levels of CRABP2 (*p* < 0.05) when compared to those of solvent controls. Treatment with 30 μM of CANA but not 10 μM noticeably reduced the protein levels of RARβ (*p* = 0.031) (Figure 9B). In contrast, CANA could remarkably reduce the levels of CRABP2 at the concentration of both 10 and 30 μM (Figure 9C). When 30 μM of MPA was combined with either 10 or 30 μM of CANA, there were significant reductions observed in the protein levels of RARβ and CRABP2 (*p* = 0.011). Additionally, no significant differences were observed in the protein levels of RARβ and CRABP2 between the treatment with CANA alone and combined with MPA (*p* > 0.05). Differently, both MPA and CANA treatment, either alone or in combination, did not affect the protein expression of RXRA when compared with the solvent treatment cells (Figure 9D).

Furthermore, the expression of BAX was also measured since CANA induced apoptosis of the THRB^(−/−)^/RL95-2 cells (Figure 9E; Supplementary Figure S6). A significant downregulation of BAX was observed after TRβ was knocked out (*p* = 0.010). Compared with that of the control cells, treatment with 30 μM of MPA alone did not obviously affect the expression of BAX in the THRB^(−/−)^/RL95-2 cells (*p* > 0.05). However, treatment with 10 and 30 μM of CANA significantly elevated the levels of BAX (*p* < 0.05) in a concentration-dependent manner. Combining CANA with MPA declined the expression of BAX but no pronounced changes were observed compared to that of treatment with CANA alone (*p* > 0.05).

Furthermore, the protein expressions of RARβ in progestin-sensitive and progestin-insensitive EAH and EC tissues were assessed through immunohistochemical analysis since RARβ was found to be highly expressed in the MPA resistant THRB^(−/−)^/RL95-2 cells. The staining of RARβ protein was mainly localized in the cytoplasm and some localized in the nucleus, demonstrating yellow or brown colors. The ratio of the positive area for RARβ was markedly different between sensitive (383,643.34 ± 220,057.17 pixels) and insensitive tissues to progestins therapy (700,237.49 ± 182,661.03 pixels; *p* = 0.005; Figures 9F,G; Supplementary Figures S7-S10). These results demonstrated that RARβ and CRABP2 were likely as potential targets in the MPA resistant THRB^(−/−)^/RL95-2 cells. Furthermore, CANA downregulated their expression and induced apoptosis in the EC cells.

### 3.8 Interaction and reciprocal regulation of TRβ, RARβ and CRABP2

**FIGURE 10:**
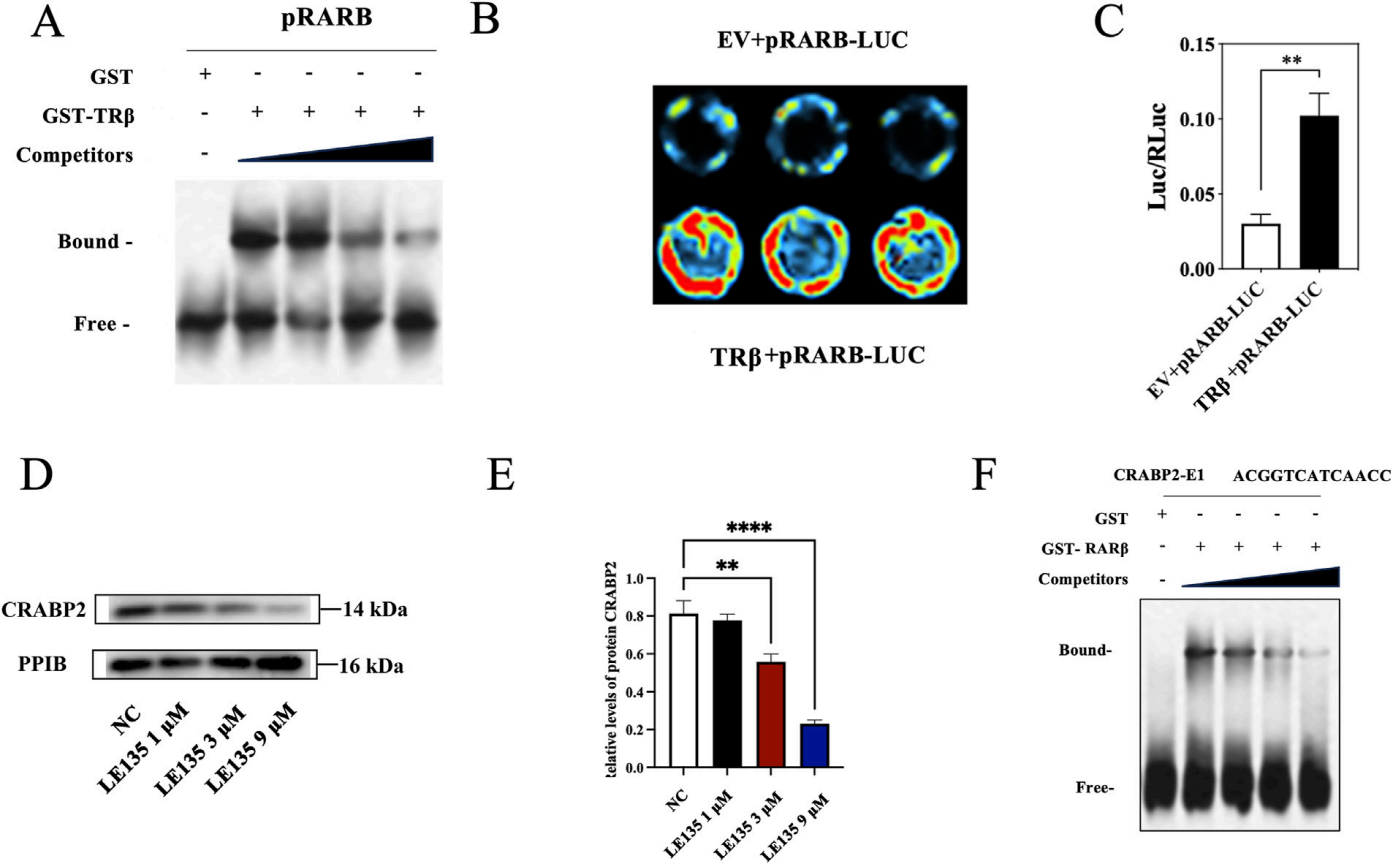
Interaction and regulation of TRβ, RARβ and CRABP2. **(A)** The interaction between TRβ and the RARβ promoter assayed by EMSA. The “bound band” was a key indicator. It represented the complex where TRβ attaches to the RARβ promoter DNA, migrating sluggishly in the gel due to the increased molecular mass from this union. In contrast, the “free band” meant the unbound RARβ promoter DNA, i.e., a smaller size protein migrated more rapidly through the gel, providing a baseline to confirm the specific binding event. **(B,C)** Relative fluorescence intensity between TRβ and RARβ promoter binding assayed by dual luciferase reporter gene. The value of Luc/RLuc was the relative fluorescence intensity. EV represented empty vector. The stronger fluorescence intensity indicated the stronger binding ability of TRβ to the RARB promoter. **(D,E)** Inhibitory effects of LE135, the RARβ inhibitor, on the expression of CRABP2 in a concentration-dependent manner. **(F)** Effects of RARβ regulated the CRABP2 promoter *via* EMSA assay. The results were presented as the mean ± SEM from three independent experiments with triplet repeat of each data. **p < 0.01, ****p < 0.0001 compared with control group.

In light of the protein levels of RARβ, RXRA and CRABP2 exhibited significant changes after the TRβ was knocked out in the RL95-2 cells, the interactions among them were further explored. In EMSA assays, when the biotin-16-UTP unlabeled RARβ promoter probes were added to bind competitively with the biotin-16-UTP labeled RARβ promoter probes, the bound band gradually weakened, indicating there was a specific binding between TRβ and the promoter of RARB (Figure 10A; Supplementary Figure S11).

In the dual luciferase reporter assay, the fluorescence signal value of the co-incubation group of TRβ and the RARβ promoter gene was markedly elevated (*p* = 0.002) when compared with that of control group, suggesting that TRβ was not only able to bind specifically to the promoter of RARβ, but also to directly regulate its expression. This finding was in line with the results of the EMSA (Figures 10B,C).

In addition, no significant changes were observed in the methylation level of the RARβ promoter before and after treatment with MPA and CANA (Supplementary Figure S14). Moreover, after treatment with LE135, the RARβ inhibitor, at the concentration of 1 and 9 μM, the protein expression of CRABP2 significantly decreased in a concentration-dependent manner in THRB^(−/−)^/RL95-2 cells (Figures 10D,E; Supplementary Figure S12), compared with those of solvent treated cells, suggesting RARβ was able to directly regulate the expression of CRABP2. Hence, the binding of RARβ and the promoter of CRABP2 was further probed with EMSA. Bound bands appeared when RARβ was conjunct to the CRABP2 promoter. When the biotin-16-UTP unlabeled CRABP2 promoter probes were added to bind competitively with the biotin-16-UTP labeled RARβ promoter probes, the bound bands gradually weakened, indicating that RARβ was able to specifically bind to the promoter of CRABP2 at the sequence -ACGGTCATCAACC- (Figure 10F; Supplementary Figure S13). These results revealed that TRβ directly regulated RARβ expression by binding to its promoter, consistent with EMSA and luciferase reporter assays. Additionally, RARβ was able to directly bind to the promoter of CRABP2 and modulate its expression.

## 4 Discussion

In this study, we established THRB^(−/−)^/RL95-2 EC cells with the gene of THRB knockout and found that the THRB^(−/−)^/RL95-2 cells exhibited resistance to progestin treatment. With a ligand-based high-throughput virtual drug screening technique, CANA was identified as a potential compound to interact with TRβ. When used in conjunction with progestin, CANA alleviated the resistance of the THRB^(−/−)^/RL95-2 cells by suppressing the proliferation of the cells and induced apoptosis. In the mice model with xenograft tumor induced by THRB^(−/−)^/RL95-2 cells, the combination of CANA (15 mg/kg) and MPA (100 mg/kg) significantly reduced the weights of the tumors compared with those of the solvent treated control mice. By using transcriptomics, proteomics and molecular dynamic techniques, the protein RARβ and CRABP2 were further identified as potential targets mediating the proliferative effects of the THRB^(−/−)^/RL95-2 EC cells, in company with significantly increased the protein levels of RARβ, CRABP2, and downregulated BAX. When adding into CANA, all expressions of the proteins were reversed whether it was used alone or combined with MPA. For the first time, we found that CANA could alleviate progestin resistance and enhance the inhibitory effects of MPA in THRB^(−/−)^/RL95-2 cells through directly targeting RARβ and modulating its downstream CRABP2 and BAX signals.

Obesity and hypercholesterolemia are currently considered as one of the key factors inducing EC (Onstad et al., 2016). TRβ served as a regulatory element of lipid metabolism, and Rezdiffra™, a novel agonist designed for this target, has been approved for the therapy of hyperlipidemia. In a previous study, we found there could be a correlation between low expression of TRβ and progestin resistance (Ren et al., 2022), presuming that supplementing with TRβ agonists could ameliorate the resistance caused by TRβ deficiency. However, adding T3, a pan-agonist of thyroid hormone receptor to the culture did not effectively inhibit the proliferation of si-THRB/RL95-2 cells, because T3 targeted both TRα and TRβ, which belonged to broadly homologous family proteins (data not shown). As we know, homologous proteins could result in poor drug selectivity due to their sequence similarity, leading to reduced efficacy (Yang et al., 2021b; Wang et al., 2024). Therefore, we turned to seek a candidate with strong ability to interact with TRβ using high-throughput virtual screening in the FDA database to exclude TRα interference. Over 3,300 FDA-approved compounds were evaluated by using the criteria of docking scores, compound-protein interaction energies and modes of action. Among them, acarbose and CANA emerged as optimal candidate compounds with the highest docking scores. Atorvastatin was also tested *in vitro* screening because it exhibited a lipid metabolism-modulating effect (Pramfalk et al., 2011; Hu et al., 2021) and highly bound with TRβ, ranking sixth in the Docking score.

Accordingly, the effects of acarbose, CANA, and atorvastatin, both individually and in combination with MPA, were assessed on several EC cell lines, including RL95-2, an estrogen receptor-positive (type I) EC cell line, and AN3CA and KLE, which belong to estrogen receptor non-responsive (type II) cell lines (Zhu et al., 2019). CANA remarkably suppressed the growth of both type I and II EC cells, with a superior inhibitory effect than atorvastatin and acarbose. As a result, CANA was screened out for further investigation.

Given that THRB silencing facilitated RL95-2 cell growth (Ren et al., 2022),the THRB^(−/−)^/RL95-2 cells with THRB knockout were established via CRISPR techniques to further investigate the role of TRβ in EC cells. After THRB was knockout, the morphology of THRB^(−/−)^/RL95-2 cells differed significantly from that of the wild type. Notably, the cells appeared smaller and more compact, with reduced intercellular gap junctions and grew in an aggregative tendency rather than as monolayers, and desmosome-like structures were observed. In the previous study, neither MPA or T3 treatment depressed the viabilities of the si-THRB/RL95-2 cells (Ren et al., 2022). Likewise, we found that MPA also failed to inhibit the growth of the THRB^(−/−)^/RL95-2 cells in the presented study. However, the inhibitory effect of MPA was partially recovered once the DNA of THRB was backfilled into the THRB^(−/−)^/RL95-2 cells, which suggested that TRβ deficiency was directly linked to dysregulated progestin sensitivity.

In THRB silenced RL95-2 cells, CANA more significantly suppressed cell viabilities than MPA. Unexpectedly, CANA was also found to reduce the viability of the THRB^(−/−)^/RL95-2 cells. It suggested that the partial inhibitory activity of CANA was independent of the expression of TRβ. Accordingly, the capabilities of proliferation and migration of the THRB^(−/−)^/RL95-2 cells were further measured. The growth of THRB^(−/−)^/RL95-2 cells was unaffected by MPA treatment alone but suppressed after CANA was added into the cultures, and the IC_50_ values were similar when using 10 or 30 μM of MPA combined with CANA, indicating that CANA was more effective in inhibiting the growth of THRB^(−/−)^/RL95-2 cells. Since both MPA and CANA displayed the optimal inhibition at the concentration of 30 μM, this concentration was selected for subsequent experiments.

Likewise, MPA treatment alone did not affect the proliferation and apoptosis in THRB^(−/−)^/RL95-2 cells when compared with the control cells. It indicated that the THRB^(−/−)^/RL95-2 cells produced antagonism to the MPA treatment. However, after treatment with 30 μM CANA, both with or without MPA, significant inhibition was detected in the proliferation and higher apoptosis and lower survival rate in both RL95-2 and THRB^(−/−)^/RL95-2 cells. These findings suggested that CANA could reduce the resistance to the MPA in the THRB knockout EC cells.

The resistance of THRB^(−/−)^/RL95-2 cells to the progestin and the inhibitory effects of CANA were further confirmed in an animal model with xenograft tumors. Tumors originating from the two types of cell transplants grew at similar rates, reaching a mean volume of 100 mm^3^ after inoculation for 7 days (RL95-2 cells) or 10 days (THRB^(−/−)^/RL95-2 cells). The growth of xenografts induced by the wild type of RL95-2 cells was aligned with our previous findings, and MPA exhibited weak inhibition on the growth of the tumor (Ma et al., 2017; Cao et al., 2019). In the presented study, the inhibitory rate of MPA on the tumor weights of THRB^(−/−)^/RL95-2 xenografts was lower than that observed in RL95-2 cells. Additionally, the body weight of the mice inoculated with THRB^(−/−)^/RL95-2 cells gradually decreased while an increase was observed in the mice loaded with RL95-2 cells. These results suggested that the xenograft tumors derived from THRB^(−/−)^/RL95-2 cells not only resisted MPA therapy but also exhibited greater malignancy than those from wild-type RL95-2. After being administered 15 mg/kg CANA to the mice, with or without MPA, the volume and weight of xenograft tumors significantly decreased whether inoculated with the wild type or THRB KO RL95-cells. The coefficients of the liver and the kidney maintained in normal ranges in all animals suggesting that no obvious toxic reactions were observed. These findings confirmed that 30 μM CANA efficiently overcame progestin resistance in the EC cells.

Currently, symptoms such as hyperglycemia, diabetes, and obesity have been found to link to the development of EC (Raffone et al., 2020; King et al., 2023) because excess fat accumulation would cause increased estrogen levels and exacerbate the proliferation of EC (King et al., 2023). The combination of metformin and MPA is being used to enhance the therapeutic effect of EC,but its specific clinical efficacy remains controversial (Mitsuhashi et al., 2016, Mitsuhashi et al., 2020; Chae-Kim et al., 2021). CANA, an SGLT-2 inhibitor, is used to treat type II diabetes and is generally considered safe and well tolerated. Theoretically, it is plausible that CANA can combine with progestins to antagonize the resistance, and we have preliminarily verified its efficacy in the present study. In addition, we did not observe any abnormal changes in the animals. All nude mice were in good condition, with normal movement and urination processes throughout the 28-day treatment. However, based on the package insert of CANA (INVOKANA^®^), the most common adverse reactions (5% or greater incidence) mainly included female genital mycotic infections and urinary tract infections, and rare severe adverse reactions involved increasing the risk of leg and foot amputations. Therefore, potential adverse effects should be carefully monitored when CANA is intended to be utilized in combination with progestins for the therapy of EC in clinic, as this is an unexplored usage. Furthermore, progestins have been reported to act as anti-inflammatory agents with immunomodulators (Fedotcheva et al., 2022). Accordingly, we presume that progestins could mitigate CANA’s side effects, but further in-depth toxicity research will be warranted in the future.

Retinoic acid receptors (RARs) and retinoid X receptors (RXRs) were nuclear receptors that specifically bind retinoic acid (RA) and function as ligand-dependent transcription factors, typically existing as dimers within cells (Schubert and Germain, 2023). RARs consisted of three subtypes: RARα, RARβ, and RARγ. Among them, RARβ plays a crucial role in regulating cancer cell differentiation, proliferation, and apoptosis (Petty et al., 2005; Li et al., 2018). RXRA could form heterodimers with various nuclear receptors, such as RAR, PPAR, and LXR (Halstead et al., 2017). In the RA signaling pathway, RXRA acted as a “universal partner,” enhancing the DNA-binding capacity and transcriptional activity of RARβ (Schubert and Germain, 2023). In endometrial cancer (EC) tissues, Tsuji et al. (2017) found that RARα expression was positively correlated with tumor grade, while RARβ expression showed an inverse relationship. Consequently, RARβ was widely regarded as a tumor suppressor in most studies. Additionally, RARβ has been linked to drug resistance in some studies (Petty et al., 2005; Ren et al., 2016; Li et al., 2018) but its association with progestin resistance remains unexplored.

In the presented study, transcriptomic and proteomic analyses revealed that RARβ and RXRA were enriched in THRB^(−/−)^/RL95-2 cells and exhibited significantly higher protein levels compared to those in wild-type RL95-2 cells, and were unaffected by 30 μM MPA treatment. In a previous article, Hu et al. (2022) found that key genes of the retinoic acid signaling pathway such as RXRA and its downstream genes, including HOX and SOX gene family, were significantly enriched in the patients with endometrial atypical hyperplasia and EC who did not respond to progestin therapy by ATAC-Seq and RNA-Seq integration analysis. Similarly, we found multiple genes associated with the retinoic acid signaling pathway were enriched in both proteomics and transcriptomics, and their expressions were validated at the protein level, leading us to select the retinoic acid signaling pathway as the focus of the study.

Electrophoretic mobility shift assay (EMSA) and dual-luciferase reporter assays demonstrated that TRβ directly bound to the RARβ promoter, indicating that TRβ directly regulated RARβ expression. As a result, we considered that THRB knockout directly activated the RA signaling pathway, consistent with the findings by Bohnsack and Kahana, (2013) in zebrafish, indicating that increased RA signaling indeed compensated for reduced TH signaling. Furthermore, Tanabe et al. (2008) have reported that RARβ was predominantly overexpressed in endometrial hyperplasia patients. Similarly, we observed significantly elevated nuclear expressions of RARβ in progestin-resistant endometrial cancer and hyperplasia tissues, particularly in progestin-resistant endometrial hyperplasia patients. In contrast, weak expressions of RARβ were generally observed in the cytoplasm of progestin-sensitive uterine tissues. These findings suggested that high RARβ expressions were likely associated with progestin resistance. However, further validations were required in larger clinical cohorts.

The results of molecular docking demonstrated that CANA bound to both RARβ and RXRA, but with a stronger binding affinity to RARβ. Molecular dynamics simulations revealed stable binding between CANA and RARβ under physiological pH conditions, mediated by π-π interactions and hydrogen bonds involving the residues PHE1230, PHE1271, PHE1288, and PHE1304 of RARβ. Treatment with 10 or 30 μM CANA induced a concentration-dependent decrease in the protein expression of RARβ and RXRA, with a more significant reduction observed in RARβ, without affecting RARβ promoter methylation levels. It indicated that CANA directly inhibited RARβ and RXRA protein expression in THRB^(−/−)^/RL95-2 cells without through epigenetic modifications.

Cellular retinoic acid-binding protein 2 (CRABP2) was a key transporter in the RA signaling pathway, responsible for delivering RA to the nucleus for binding with RARs, thereby regulating cell differentiation, proliferation, and embryonic development (Hewson et al., 2002). Recent studies demonstrated that higher expressions of CRABP2 were likely correlated to poor prognosis and tumor progression in various cancers, including glioblastoma, EC, breast cancer, and non-small cell lung cancer, and therefore regarded as a potential biomarker for endometrial cancer malignancy (Wu et al., 2019; Egan et al., 2022; Liu et al., 2022; Fu et al., 2024). Furthermore, CRABP2 overexpression has been linked to chemotherapy resistance in both ovarian and breast cancers (Zeng et al., 2023; Fu et al., 2024). In the presented study, we found that CRABP2 was enriched in both transcriptomics and proteomics in the THRB^(−/−)^/RL95-2 cells, and its protein expression was significantly elevated but unaffected by 30 μM MPA treatment, suggesting CRABP2 likely plays a role in progestin resistance. Treatment with the RARβ inhibitor LE135 led to a concentration-dependent reduction in CRABP2 protein levels (Li et al., 1999). Moreover, the result of EMSA demonstrated that RARβ, as a transcription factor, could bind to the CRABP2 promoter at the sequence -ACGGTCATCAACC-. Those results indicated that RARβ not only bound to the transporter of the retinoic acid signaling pathway but also directly modulated its expression. In addition, we found that CANA treatment markedly suppressed the protein levels of RARβ and CRABP2 in a concentration-dependent manner; however, molecular docking revealed no direct binding between CANA and CRABP2, implying that the inhibition of CANA on CRABP2 was mediated through RARβ. Taken together, these findings suggested that CANA counteracted MPA resistance were likely *via* inhibiting the expression of CRABP2 and promoting apoptosis in THRB^(−/−)^/RL95-2 cells.

In addition, we observed CANA enhanced the expression of BAX and facilitated apoptosis, but no binding sites between CANA and BAX were identified. Both Tang (Tang et al., 2022) and Zhang et al. (2021) have reported that CRABP2 has the function of promoting the ubiquitinated degradation of BAX. Given the established association between BAX and CARBP2, it is plausible that one of the mechanisms by which CANA upregulates BAX involves suppression of RARβ/CRABP2. The increased expression of BAX facilitates cell apoptosis, which would contribute to alleviating progestin resistance. It would be further validated in the future experiment.

## 5 Conclusion and limitation

**FIGURE 11:**
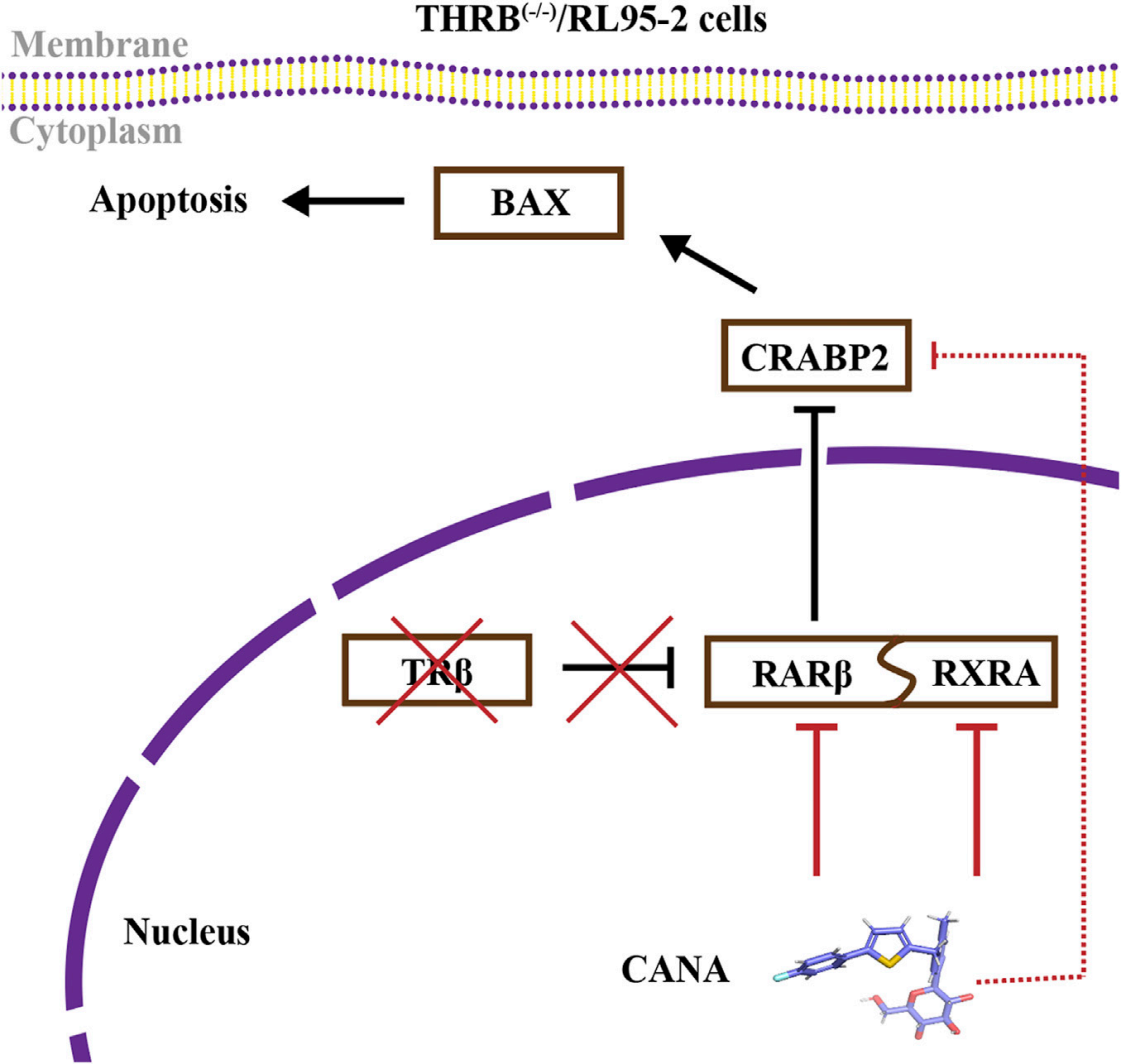
CANA alleviated progestin resistance and induced cell apoptosis by impeding the RA signaling pathway in THRB^(−/−)^/RL95-2 cells.

In the presented study, we demonstrated that TRβ deficiency was likely leading to progestin resistance via interfering with RA signaling pathway in EC cells. CANA significantly induced apoptosis of THRB^(−/−)^/RL95-2 cells through directly targeting RARβ and RXRA, accompanied by suppressing the expression of CRABP2 and restoring BAX levels, resulting in overcoming progestin resistance. Combining CANA and MPA would offer a novel strategy for improving sensitivity and efficacy for progestin therapy in clinical settings (Figure 11). Here, we explored the preliminary mechanisms by which the RA signaling pathway induces EC progestin resistance and demonstrated the potential therapeutic efficacy of CANA. Nevertheless, the study had several limitations. The sample size of the uterine tissues used for RARβ detection were small and should be expanded in future clinical studies. In addition, further investigation is needed to understand the impact of RARβ on EC prognosis, as well as its relationship with other receptors, such as estrogen and progesterone receptors. The mechanisms by which CANA targets RARβ, CRABP2, and BAX also require further validation through additional *in vivo* and *in vitro* experiments. Furthermore, the therapeutic effect of CANA has yet to be warranted in patient-derived models and clinical practice.

## Data Availability

The datasets presented in this study can be found in online repositories. The names of the repository/repositories and accession number(s) can be found below: https://www.ncbi.nlm.nih.gov/, PRJNA1220596 http://www.proteomexchange.org/, PXD060591.

## Glossary

AC: Adenocarcinoma;
APS: Ammonium persulfate
ATCC: American Type Culture Collection
BAX: Bcl-2-associated X Protein
CANA: Canagliflozin
CCK-8: Cell Counting Kit-8
CR: Complete Response
CRABP2: Cellular Retinoic Acid-binding Protein 2
Co-IP: Co-immunoprecipitation
DEG: Differentially Expressed Gene
DEP: Differentially Expressed Protein
DIA: Data-Independent Acquisition
DMEM: Dulbecco’s Modified Eagle Medium
DMSO: Dimethyl sulfoxide
EAH: Endometrial Atypical Hyperplasia
EC: Endometrial Cancer
FBS: Fetal Bovine Serum
GO: Gene Ontology
HBSS: Hank’s Balanced Salt Solution
IHC: Immunohistochemistry
IP: immunoprecipitation
KEGG: Kyoto Encyclopedia of Genes and Genomes
KO: knockout
LUC: Luciferase
MA: Mgestrol Acetate
MD: Molecular Dynamics
MolSA: Molecular Surface Area
MPA: Medroxyprogesterone Acetate
MM-GBSA: Molecular Mechanics/Generalized Born Surface Area
OD: Optical Density
OM: Optical Microscope
PVDF: Polyvinylidene Difluoride
PSA: Polar Surface Area
RA: retinoic acid
RARB: retinoic acid receptor B
RARβ: retinoic acid receptor β
RLUC: Renilla Luciferase
RXRA: Retinoid X Receptor α
rGyr: radius of Gyration
RIPA: Radioimmunoprecipitation Assay
SDS: Sodium Dodecyl Sulfate
SGLT2: Sodium-Glucose Cotransporter 2
T3: Triiodothyronine
TEM: Transmission Electron Microscope
TEMED: Tetramethylethylenediamine
THRA: Thyroid Hormone Receptor A
THRB: Thyroid Hormone Receptor B
TRα: Thyroid Hormone Receptor α
TRβ: Thyroid Hormone Receptor β;
