# Supplementary figures and images for: Canagliflozin alleviates progestin resistance by suppressing RARβ/CRABP2 signaling in THRB knockout endometrial cancer cells
Source: Front Pharmacol. 2025 Apr 30;16:1573032. doi: 10.3389/fphar.2025.1573032 (PMC12075957; doi:10.3389/fphar.2025.1573032)

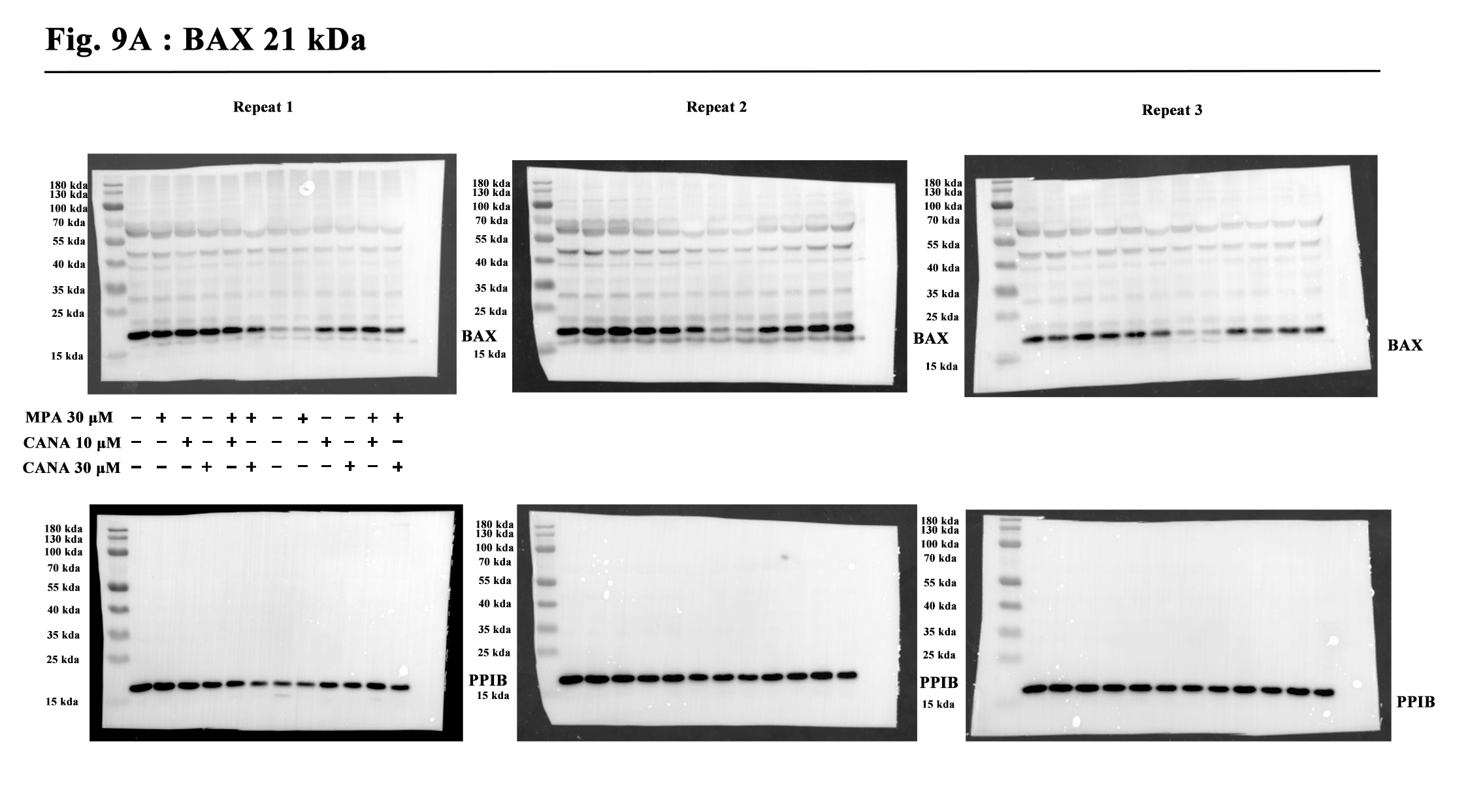

Supplement: Supplementary file 1 [file Image6.tif]

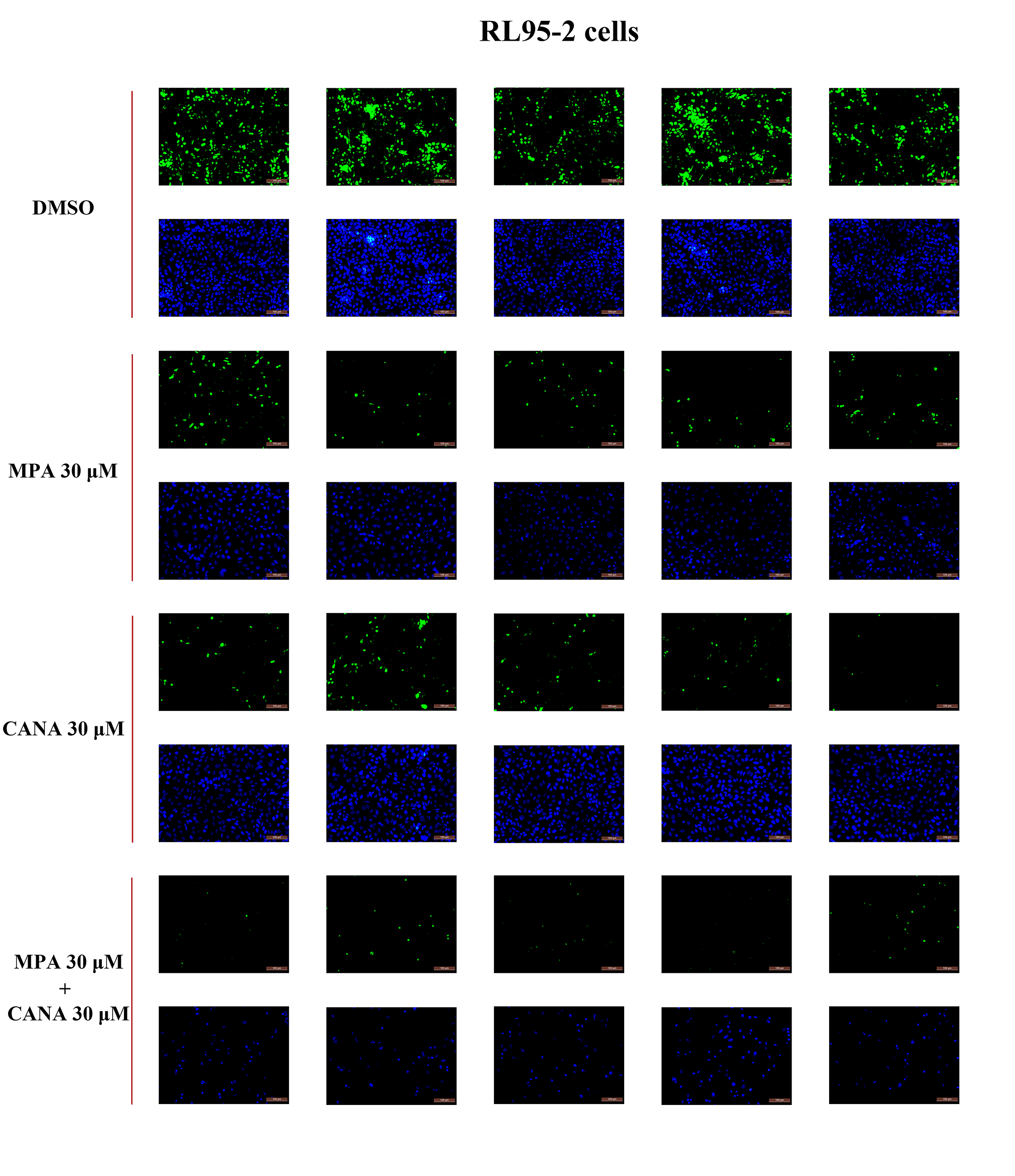

Supplement: Supplementary file 4 [file Image3.tif]

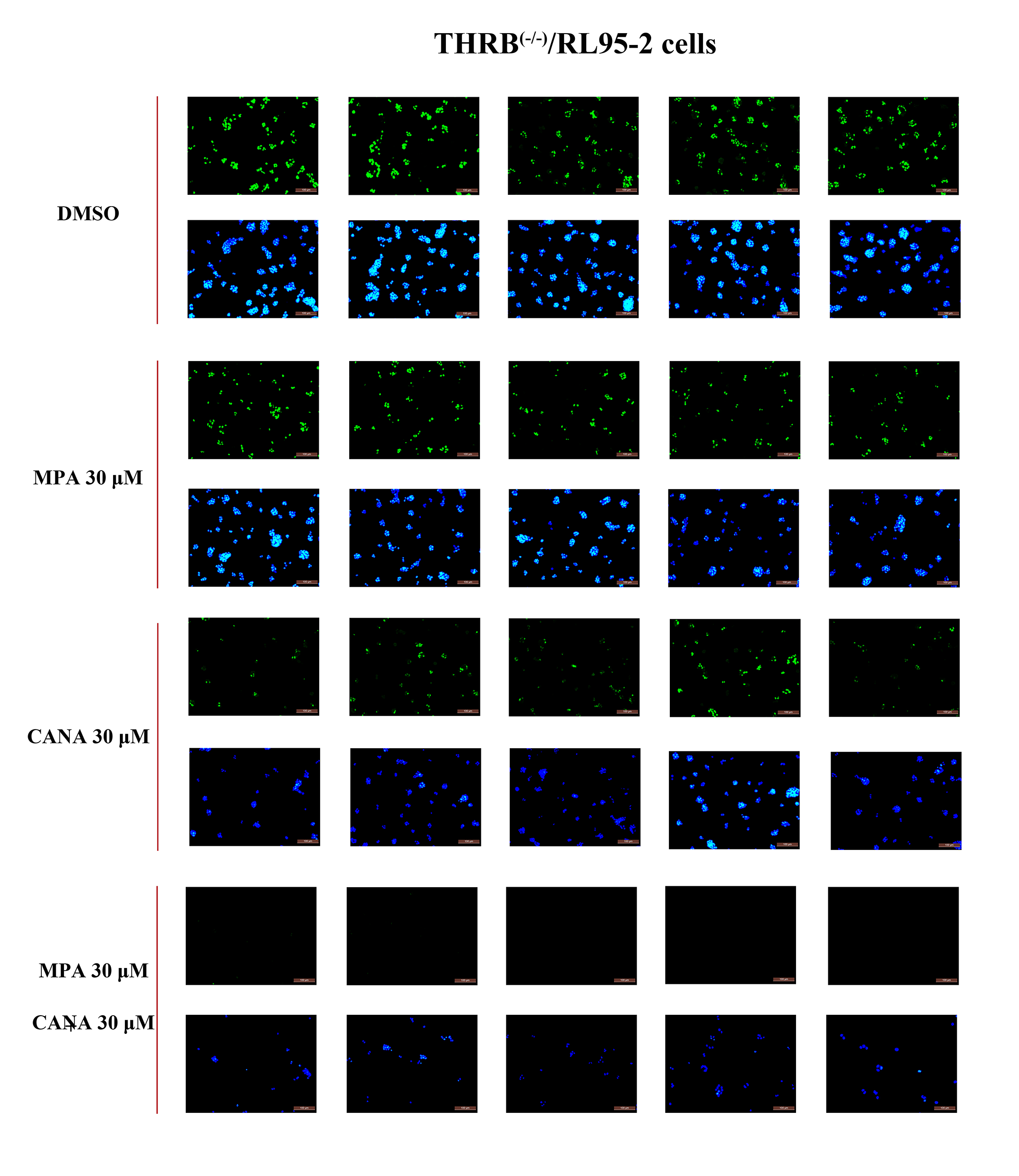

Supplement: Supplementary file 5 [file Image4.tif]

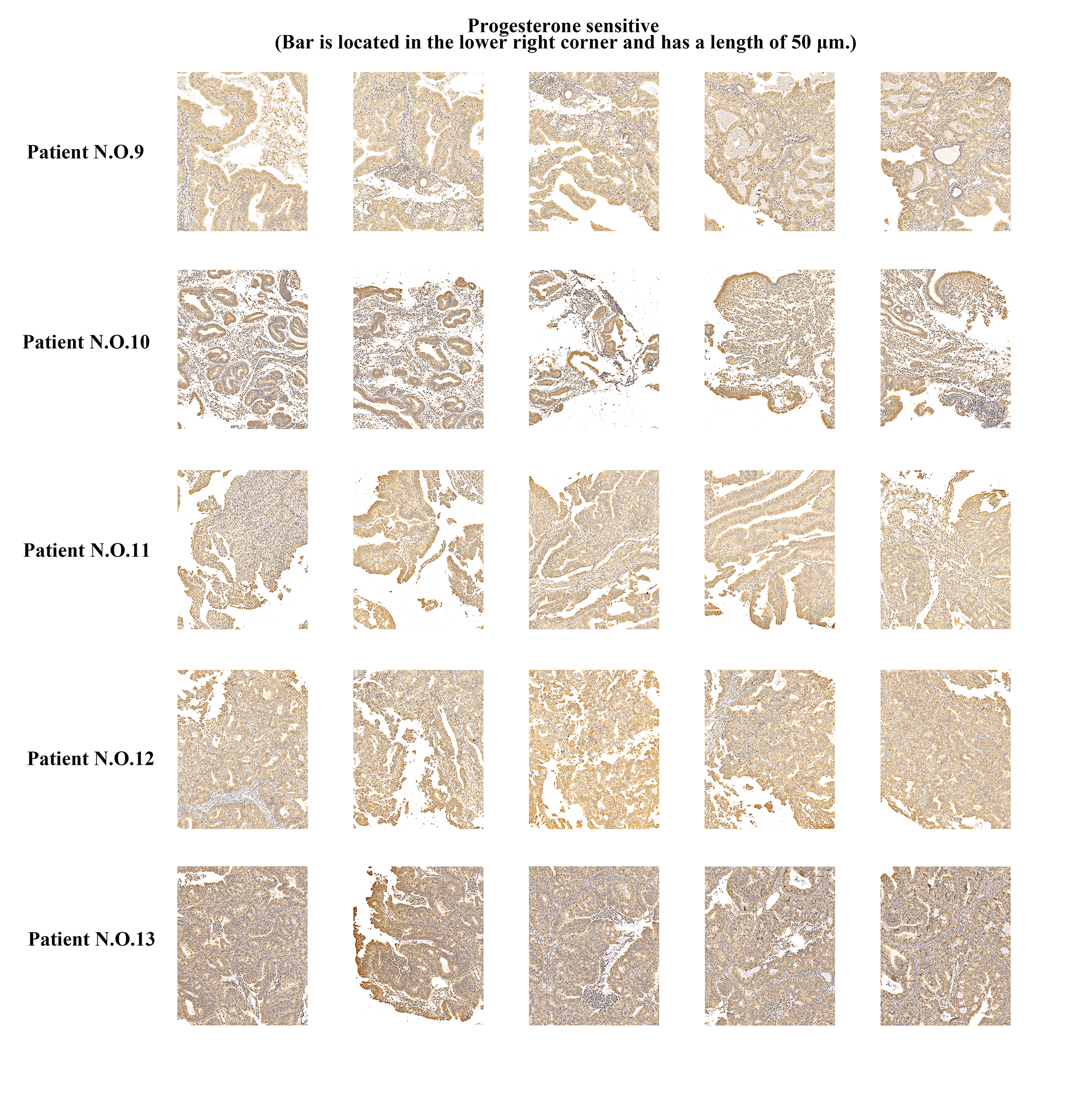

Supplement: Supplementary file 6 [file Image9.tif]

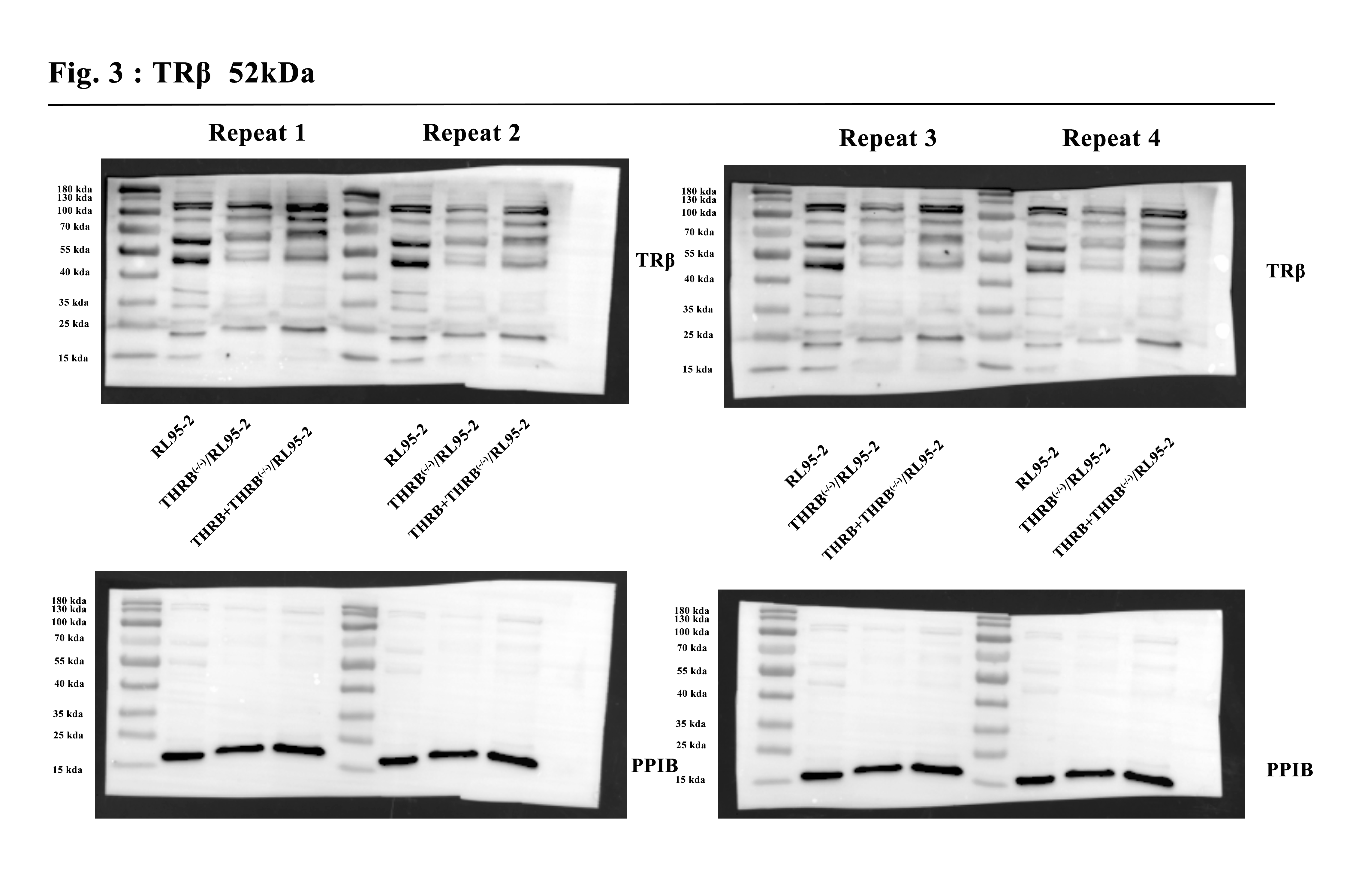

Supplement: Supplementary file 7 [file Image2.tif]

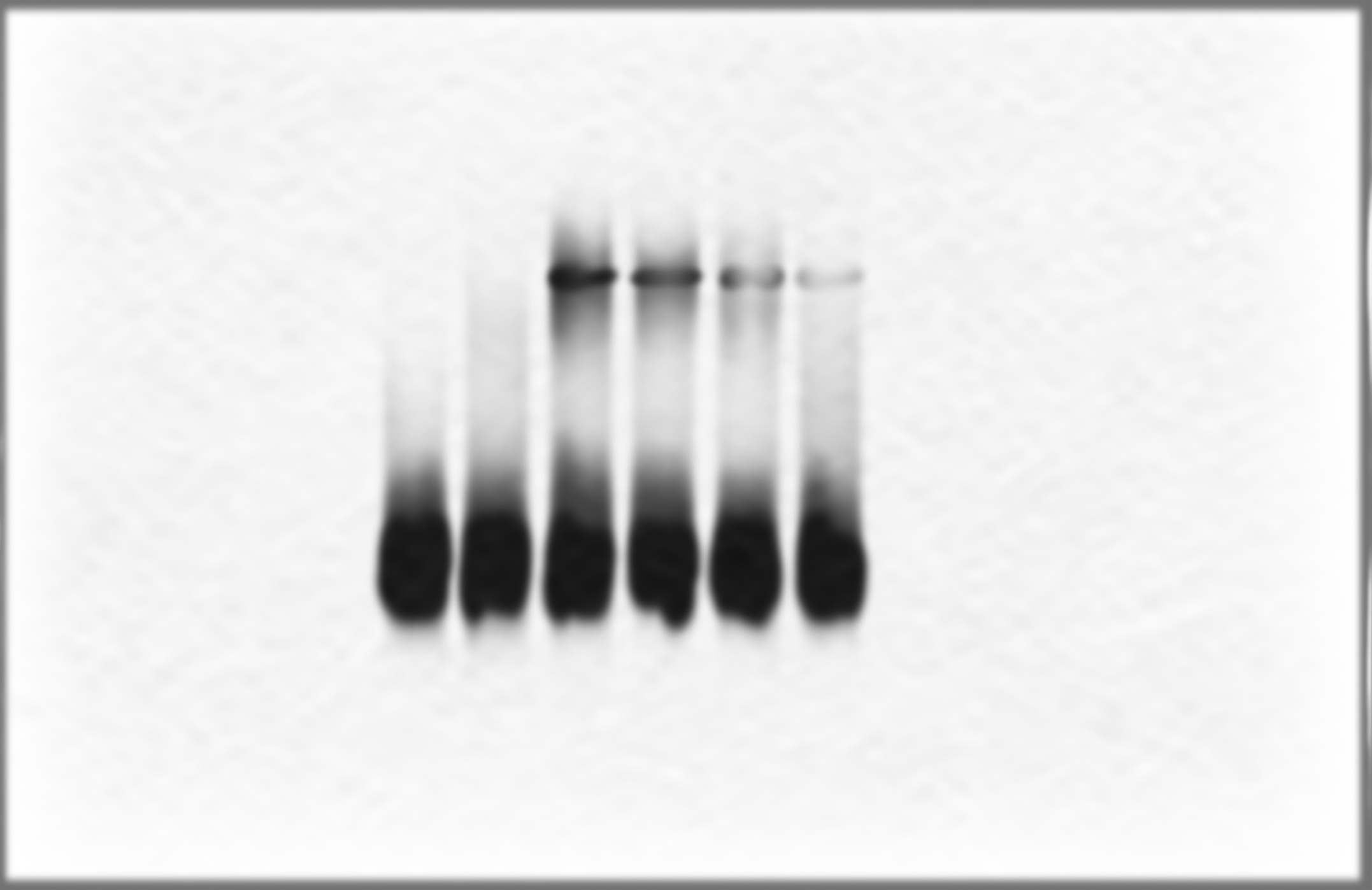

Supplement: Supplementary file 8 [file Image13.tif]

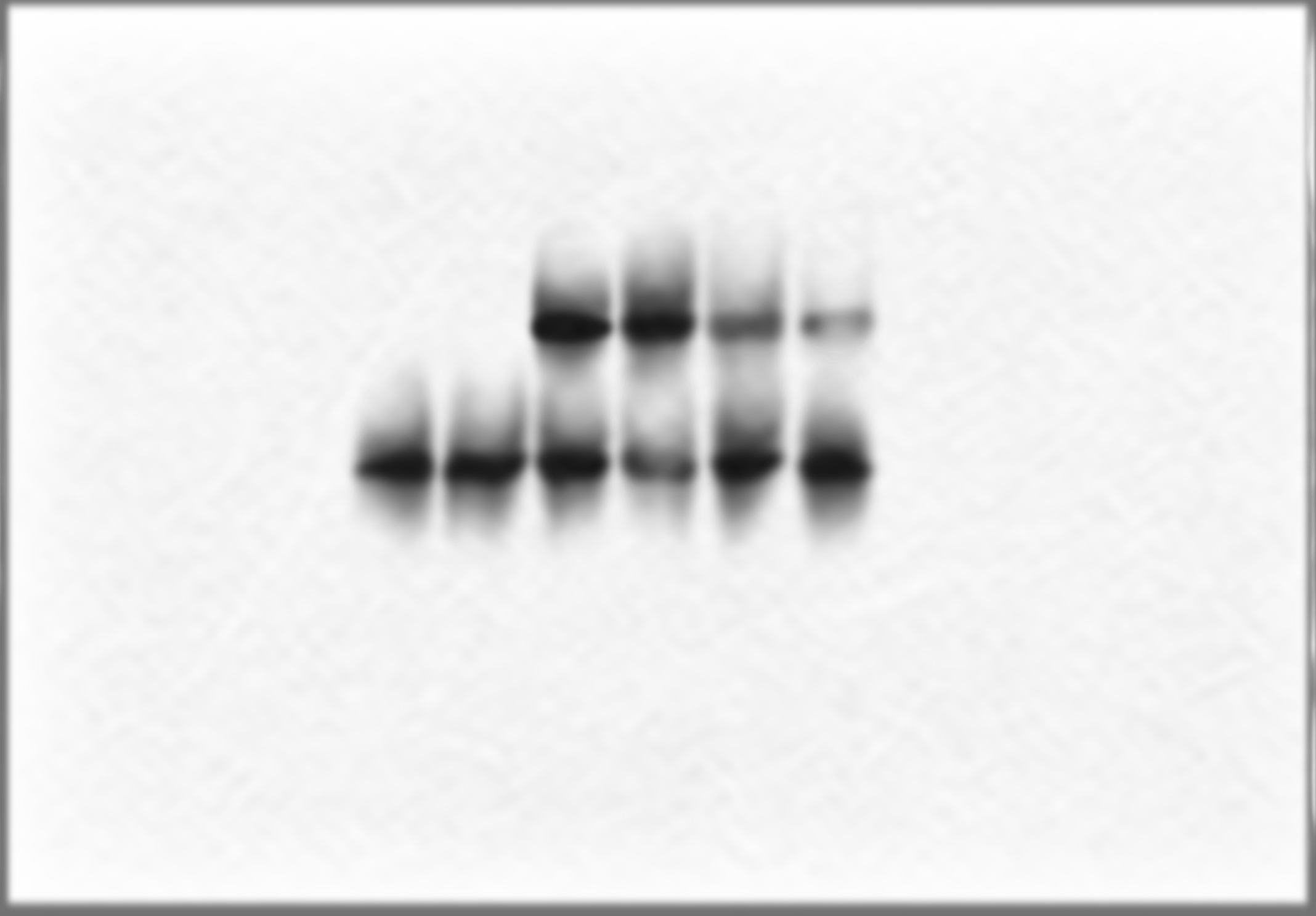

Supplement: Supplementary file 9 [file Image11.tif]

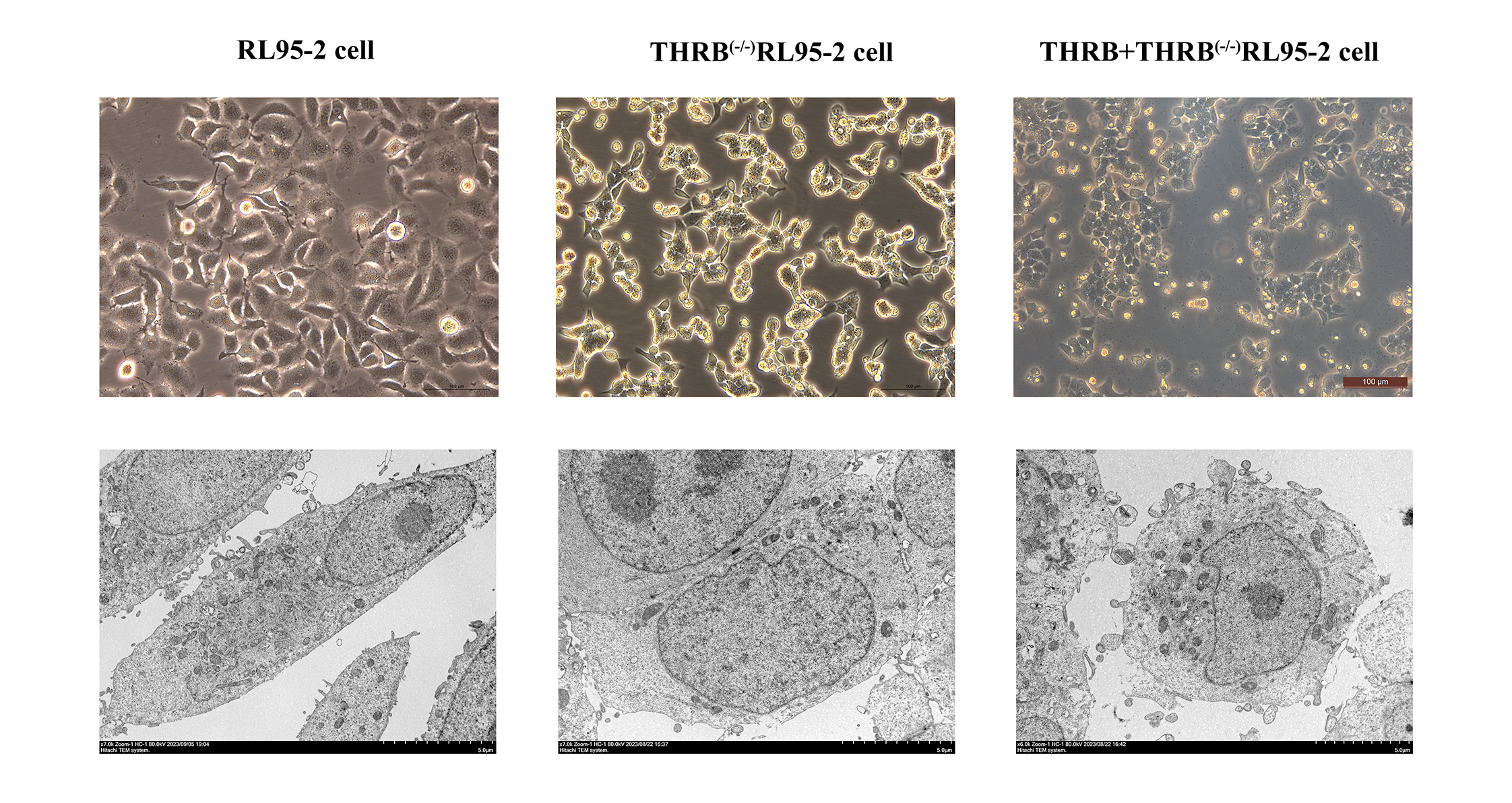

Supplement: Supplementary file 10 [file Image1.tif]

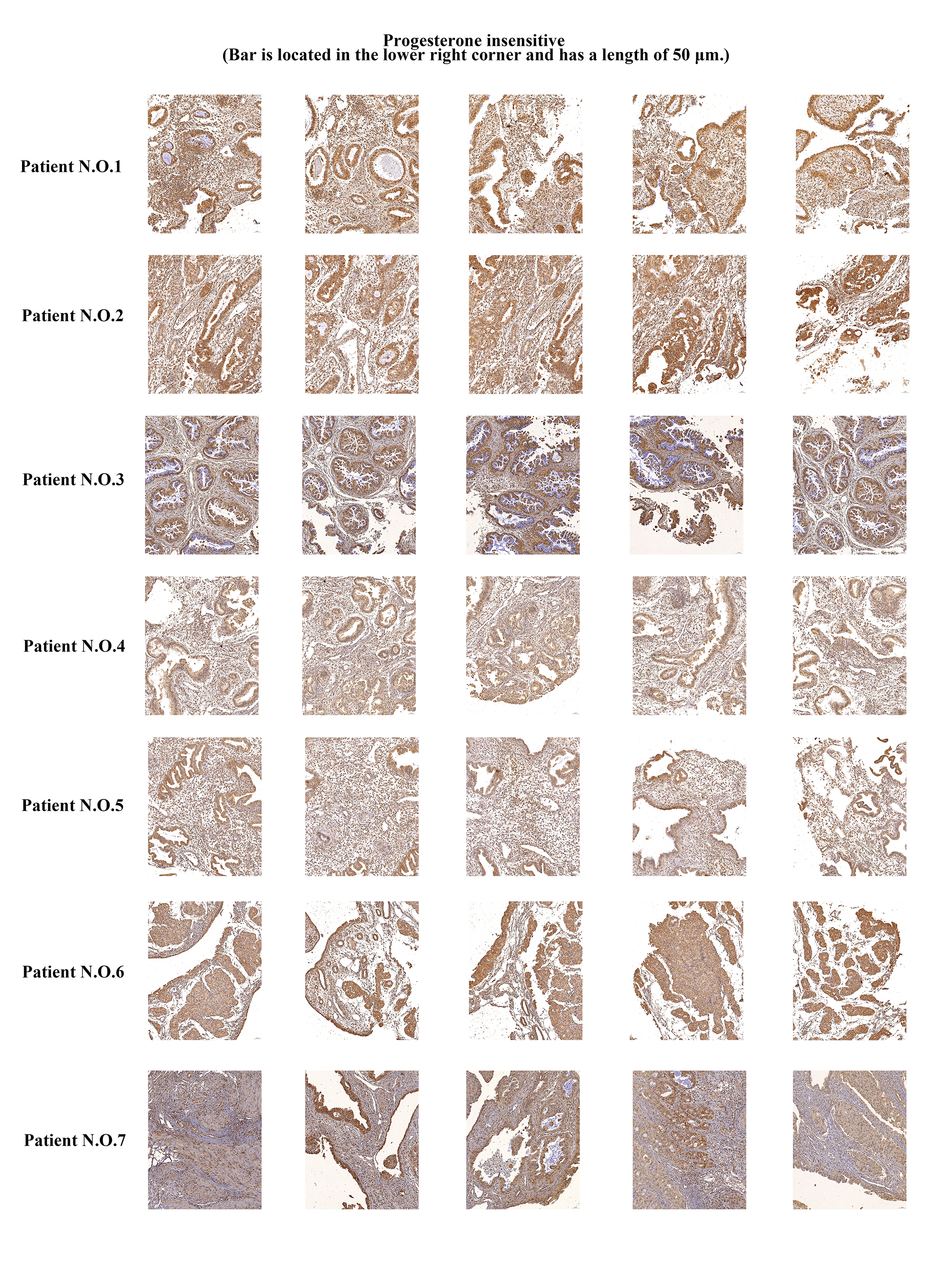

Supplement: Supplementary file 11 [file Image10.tif]

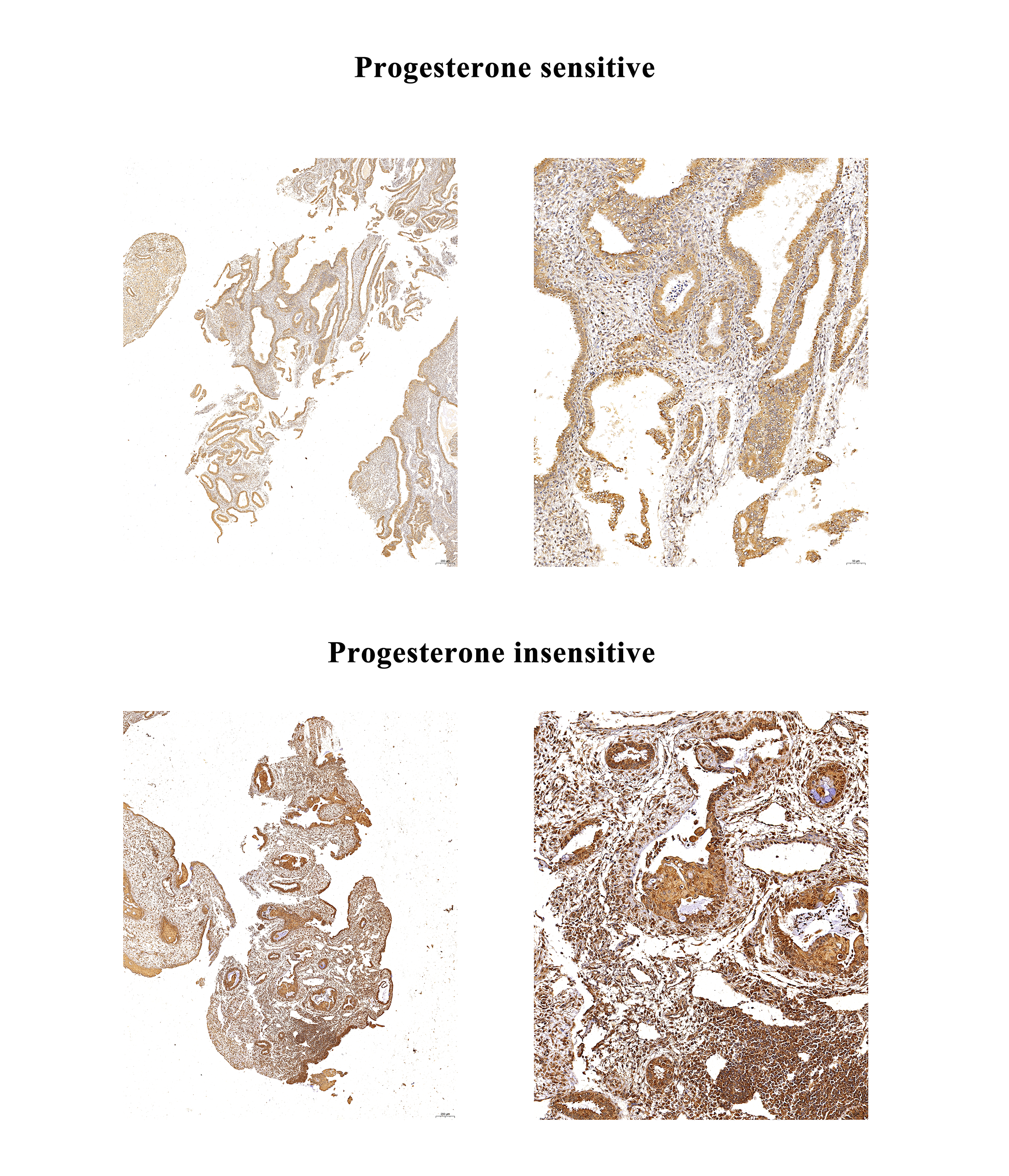

Supplement: Supplementary file 12 [file Image7.tif]

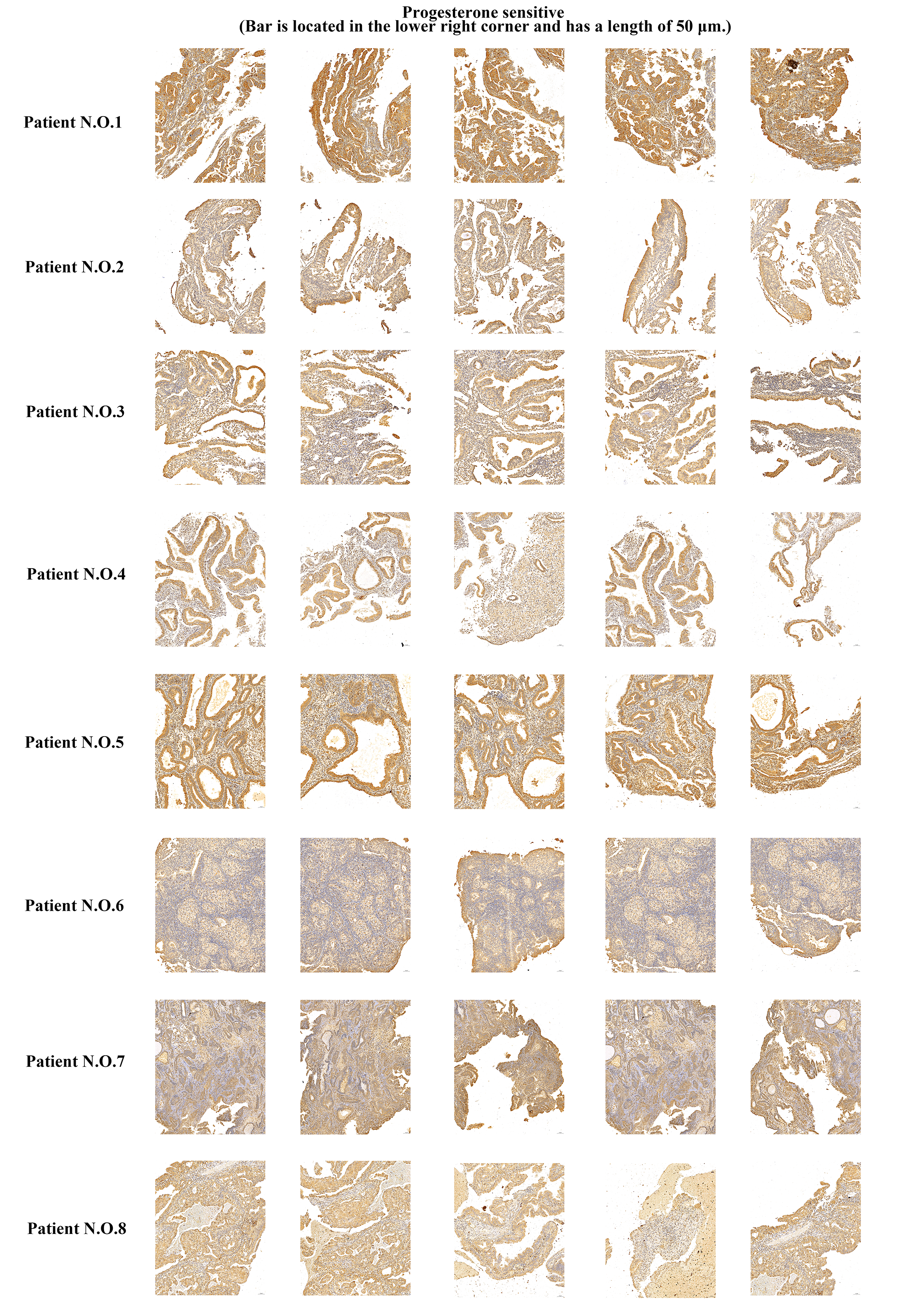

Supplement: Supplementary file 13 [file Image8.tif]

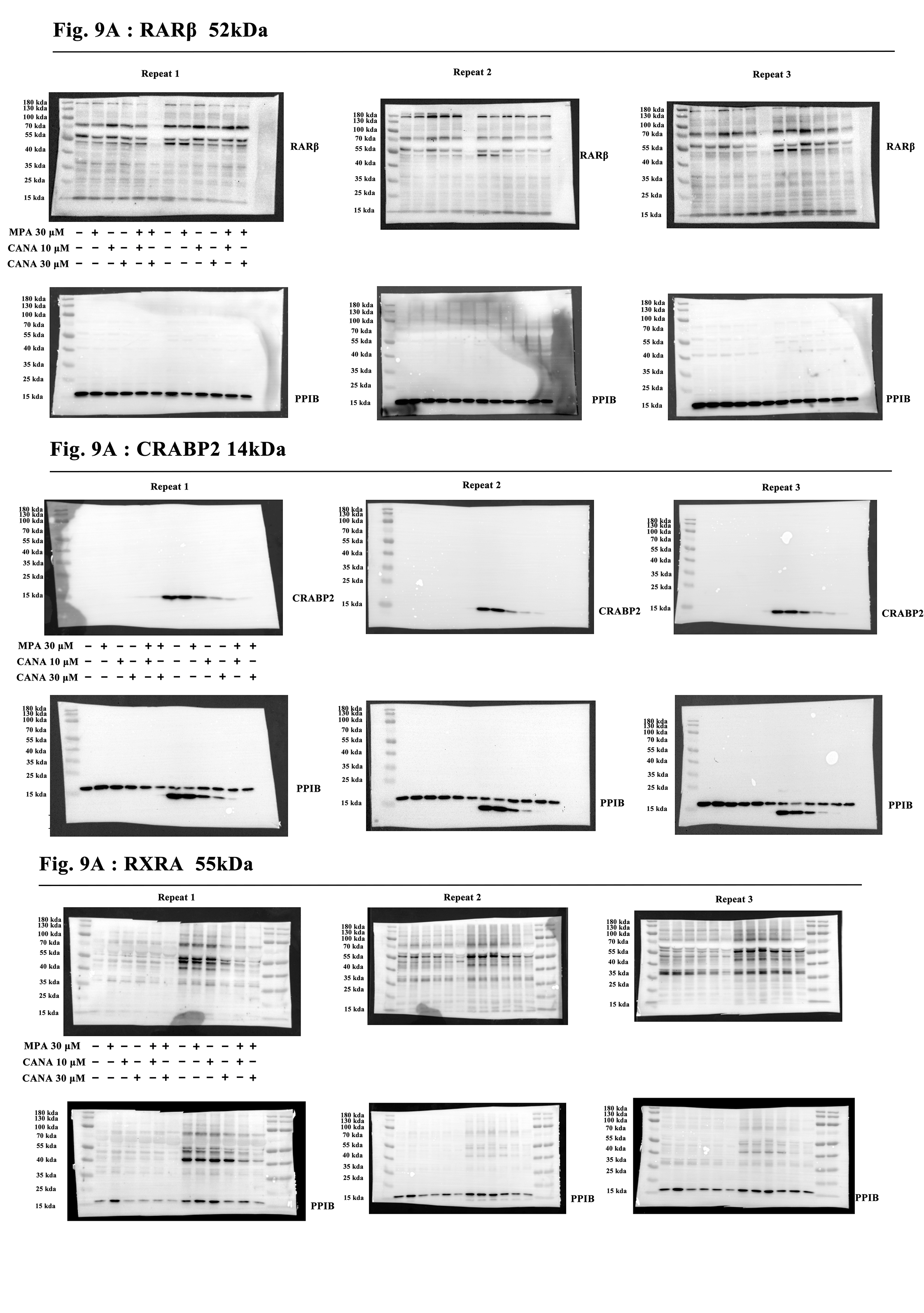

Supplement: Supplementary file 14 [file Image5.tif]

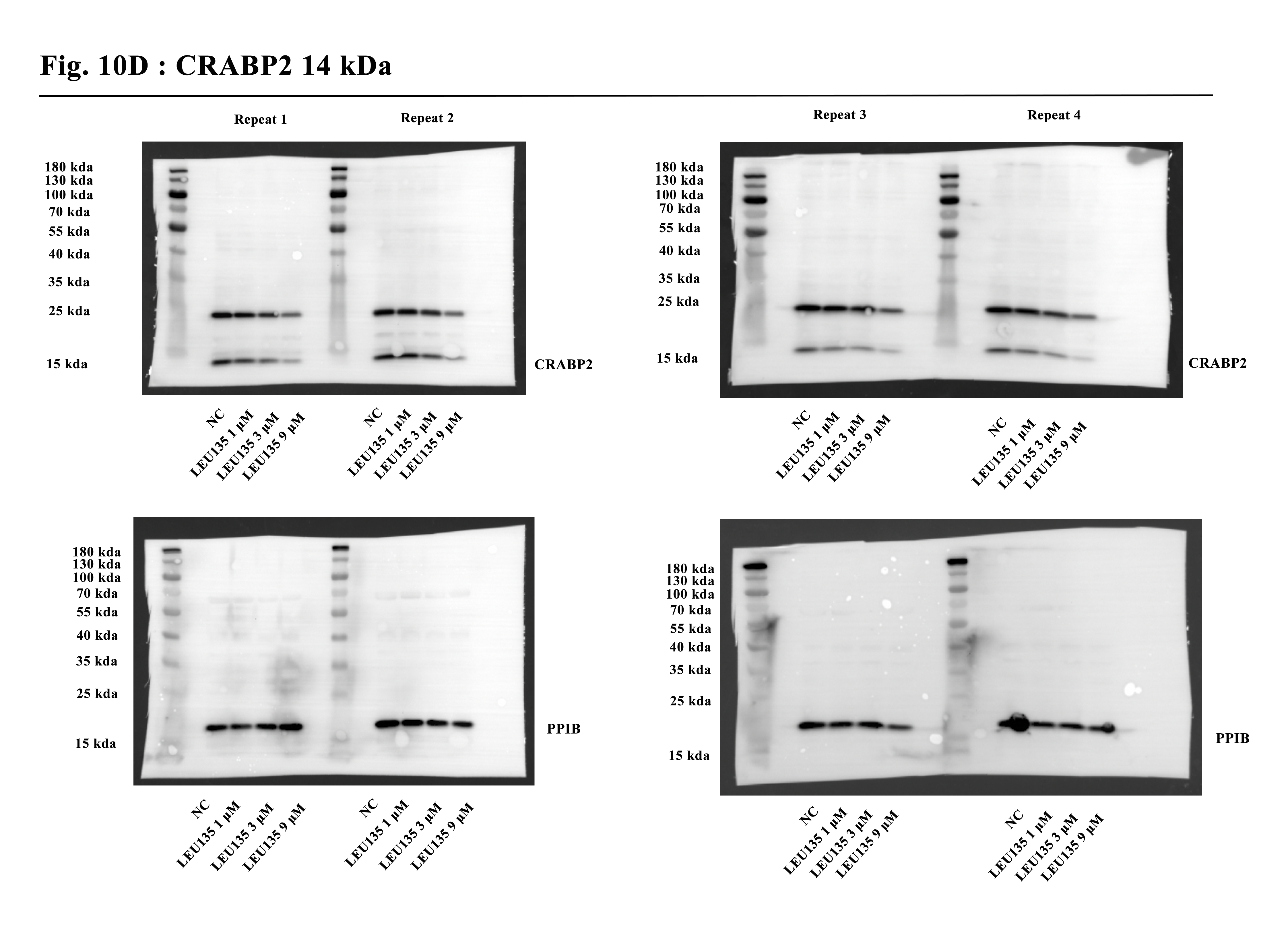

Supplement: Supplementary file 15 [file Image12.tif]
