# Supplementary material for: Canagliflozin alleviates progestin resistance by suppressing RARβ/CRABP2 signaling in THRB knockout endometrial cancer cells
Source: Front Pharmacol. 2025 Apr 30;16:1573032. doi: 10.3389/fphar.2025.1573032 (PMC12075957; doi:10.3389/fphar.2025.1573032)
Supplement: Supplementary file 3 [file Presentation1.pdf]

Figure S1：Raw optical and transmission electron microscopy images showing the morphology of RL95-2, THRB$^{(-/-)}$/RL95-2, and THRB+THRB$^{(-/-)}$/RL95-2 cells, corresponding to Figure 3A.

Figure S2：Raw western blotting data for TRβ corresponding to Figure 3B.

Figure S3：Raw data images of EdU-positive RL95-2 cells ratio of Figure 5A.

Figure S4：Raw data images of EdU-positive THRB$^{(-/-)}$/RL95-2 cells ratio of Figure 5A.

Figure S5：Raw western blotting data for RARβ, CRABP2, and RXRA corresponding to Figure 9A.

Figure S6: Raw western blotting data for BAX corresponding to Figure 9A.

Figure S7: Raw data plot Part I of RARβ immunohistochemical analysis and differential protein expression statistics in progestin-sensitive (n = 13) and progestin-insensitive (n = 7) uterine tissues, corresponding to Figure 9F.

Figure S8: Raw data plot Part II of RARβ immunohistochemical analysis and differential protein expression statistics in progestin-sensitive (n = 13) and progestin-

insensitive (n = 7) uterine tissues, corresponding to Figure 9F.

Figure S9: Raw data plot Part III of RARβ immunohistochemical analysis and differential protein expression statistics in progestin-sensitive (n = 13) and progestin-insensitive (n = 7) uterine tissues, corresponding to Figure 9F.

Figure S10：Raw data plot Part IV of RARβ immunohistochemical analysis and differential protein expression statistics in progestin-sensitive (n = 13) and progestin-insensitive (n = 7) uterine tissues, corresponding to Figure 9F.

Figure S11：Raw data of the interaction between TRβ and the RARβ promoter as assessed by EMSA in Figure 10A.

Figure S12: Raw western blotting data for CRABP2 corresponding to Figure 10D.

Figure S13: Raw data of the interaction between RARβ and the CRABP2 promoter as assessed by EMSA in Figure 10F.

Figure S14: RARB promoter methylation levels. **(A-F)** RL95-2 was treated with DMSO, 30 μM MPA 30, 10 μM CANA, 30 μM CANA, 10 μM CANA plus 30 μM MPA, and 30 μM CANA plus 30 μM MPA, respectively, and RARβ promoter methylation level changes. **(G-L)** THRB$^{(-/-)}$/RL95-2 was treated with DMSO, 30 μM

MPA 30, 10 μM CANA, 30 μM CANA, 10 μM CANA plus 30 μM MPA, and 30 μM CANA plus 30 μM MPA, respectively, and RARβ promoter methylation level changes. **(M)** RARB promoter methylation level statistics. The results were presented as the mean ± SEM from five independent experiments of each data.
